# Follicular fluid composition and reproductive outcomes of women with polycystic ovary syndrome undergoing *in vitro* fertilization: A systematic review

**DOI:** 10.1007/s11154-023-09819-z

**Published:** 2023-07-26

**Authors:** Mafalda V. Moreira, Emídio Vale-Fernandes, Inês C. Albergaria, Marco G. Alves, Mariana P. Monteiro

**Affiliations:** 1 ICBAS - School of Medicine and Biomedical Sciences, UMIB - Unit for Multidisciplinary Research in Biomedicine, University of Porto, Rua Jorge Viterbo Ferreira, Porto, 228 4050-313 Portugal; 2 ITR- Laboratory for Integrative, Translational Research in Population Health, Porto, Portugal; 3 i3S - Instituto de Investigação e Inovação em Saúde, Universidade do Porto, Porto, Portugal; 4 Centre for Medically Assisted Procreation / Public Gamete Bank, Gynaecology Department, Centro Materno-Infantil do Norte Dr. Albino Aroso (CMIN), Centro Hospitalar Universitário de Santo António (CHUdSA), 4099-001 Porto, Portugal

**Keywords:** Polycystic ovary syndrome, Assisted reproductive technology, Follicular fluid, Biomarkers

## Abstract

Polycystic ovary syndrome (PCOS) is recognized as one of the most prevalent endocrinopathy in women at reproductive age. As affected women tend to have poorer assisted reproductive technology (ART) outcomes, PCOS has been suggested to endanger oocyte quality and competence development. The aim of this systematic review was to summarize the available evidence on how the follicular fluid (FF) profile of women with PCOS undergoing *in vitro* fertilization (IVF) treatment differs from the FF of normo-ovulatory women. For that, an electronic search in PubMed and Web of Science databases was conducted (up to December 2021). The Preferred Reporting Items for Systematic Reviews and Meta-Analyses - PRISMA guidelines were followed, and the Newcastle-Ottawa Scale was used to assess the risk of bias in the included studies. Data retrieved from papers included (n=42), revealed that the FF composition of women with PCOS compared to those without PCOS predominantly diverged at the following molecular classes: oxidative stress, inflammatory biomarkers, growth factors and hormones. Among those biomarkers, some were proposed as being closely related to pathophysiological processes, strengthening the hypothesis that low-grade inflammation and oxidative stress play a critical role in the pathogenesis of PCOS. Notwithstanding, it should be noticed that the available data on PCOS FF fingerprints derives from a limited number of studies conducted in a relatively small number of subjects. Furthermore, phenotypic heterogeneity of PCOS hampers wider comparisons and weakens putative conclusions. Therefore, future studies should be focused at comparing well characterized patient subgroups according to phenotypes.

## Introduction

Polycystic ovary syndrome (PCOS) is one of the most common endocrine disorders that affects approximately 15 to 20% of women of reproductive age [1], which is accountable for up to 80% of the causes of anovulation and decreased female fertility [2]. PCOS contemplates multiple pathological mechanisms, in which the interaction between genetic and environmental factors appears to be involved, although the exact aetiology remains elusive [3]. Despite the heterogeneity of manifestations, PCOS typical clinical features include hyperandrogenism and ovarian dysfunction. The hallmarks of ovarian dysfunction include follicular development arrest, ovaries with multiple microcysts and anovulation, which can lead to decreased fertility [4].

Due to the disorder's complexity and the uncertain aetiology, PCOS treatment is targeted towards mitigating patients’ symptoms, namely the clinical signs of hyperandrogenism and/or anovulation leading to infertility. According to the international evidence-based guideline for the Assessment and Management of PCOS, anovulatory infertility can be managed with ovulation induction therapies, including first- and second-line drugs (i.e., letrozole, clomiphene citrate, and gonadotropins, used alone or in combination), while ovarian stimulation for assisted reproductive technology (ART) with *in vitro* fertilization (IVF) can be considered as a third-line intervention in case of failure of the former [5]. However, despite women with PCOS undergoing ovarian stimulation for IVF usually being good responders, with a higher than average number of growing ovarian follicles, the reproductive outcomes of these women tend to be poorer when compared to those with other causes of infertility [6–9]. Therefore, additional factors related to oocyte quality [10], as well as endometrial competence [11] have been proposed as potentially implicated in impairing the reproductive outcomes of women with PCOS. Hence, PCOS has been hypothesized to endanger the follicular microenvironment compromising oocyte’s physiological functions [12, 13].

The follicular fluid (FF) is a biological fluid present in growing secondary follicles that consists of a plasma exudate enriched in secretory products from granulosa cells (GCs) and thecal cells [14]. There is a tight relationship between the oocyte and the surrounding GCs through the FF microenvironment, which is a via for nutrient exchange and biological signal transmission, hence the importance of the FF microenvironment for follicle development and maturation [15, 16]. Notwithstanding, as consequence of the intimate contact between the FF and the cumulus-oocyte complex, FF’s profiling harbours the potential to unveil molecular signatures and biomarkers that could pinpoint to the mechanisms of disease and predict oocyte quality and reproductive outcomes of women in general, and specifically those with PCOS. Indeed, FF of women with PCOS assessed after oocyte retrieval for IVF treatment, was recently found to depict a considerably different molecular composition when compared to the FF of unaffected women [17, 18]. Nevertheless, how each molecule can potentially contribute for creating an adverse environment to the developing oocyte and in which extent it affects the reproductive outcomes of women with PCOS, has not yet been fully elucidated.

Thus, the aim of this systematic review was to summarize the available evidence on how the FF molecular profile of women with PCOS undergoing IVF treatment differs from the FF of unaffected women. Our goal was also to identify which of the identified biomarkers were demonstrated to correlate with oocyte competence and, subsequently could be useful to predict reproductive outcomes.

## Methods

### Protocol and registration

The present systematic review was conducted in accordance with Preferred Reporting Items for Systematic reviews and Meta-Analyses (PRISMA) 2020 statement [19]. The protocol was registered at inception on February 16^th^, 2021 in the international database of prospectively registered systematic reviews (PROSPERO) [20] and can be accessed with the registration number CRD42021237734.

### Information sources and search approach

An electronic search in PubMed and Web of Science databases was conducted for reports published up to December 2021, using a search strategy based on the following concepts: polycystic ovarian syndrome, follicular fluid, *in vitro* fertilization, and reproductive outcomes related to oocyte-embryo development. Medical Subject Headings in PubMed and synonyms were used to outspread the search. No restrictions regarding language and publication year or other filters were used.

### Study design and selection criteria

**Fig. 1:**
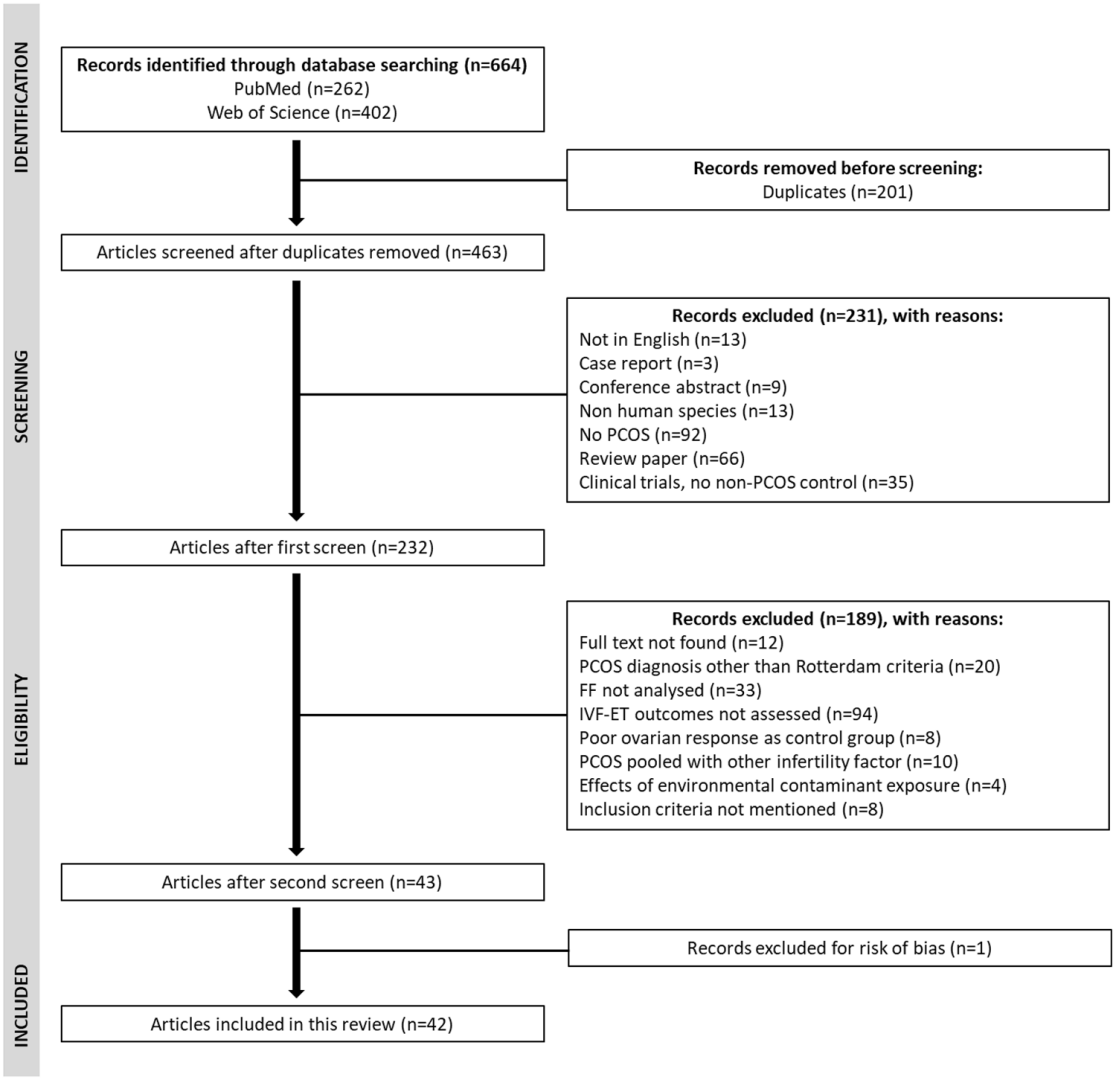
Flowchart of search and selection process for studies included in the review, adapted from PRISMA 2020 flow diagram. FF = Follicular Fluid, IVF-ET = *In Vitro* Fertilization – Embryo Transfer, PCOS = Polycystic Ovary Syndrome

After removing duplicates, the study selection process consisted in a stepwise approach by eliminating non-eligible studies based on the information provided in the abstract, followed by full text analysis of studies considered potentially eligible in the first phase as described in further detail in Fig. 1. The eligibility inclusion criteria were based on PICOS (Population, Intervention, Control intervention, Outcome, Study design) and included observational studies (cohort and case-control studies); studies conducted in humans, irrespective of race and with no restrictions on population size; studies with at least one group including women with normal ovarian function and another group comprising women with PCOS diagnosis. The exclusion criteria included papers written in languages other than English or Portuguese; studies whose full text could not be accessed; grey literature; abstracts; reviews or position statement papers; studies in which PCOS diagnosis was not established based on Rotterdam criteria; studies that did not provide information on participants’ diagnosis and inclusion and exclusion criteria; studies with pooled data pertaining to women with PCOS and other conditions associated with impaired ovarian function (e.g., endometriosis); studies that included women with conditions that could potentially impair ovarian function as control group; studies that analysed serum and/or plasma or embryo culture medium or follicular cells but not FF and; studies that did not assess the relationship between FF composition and IVF outcomes.

### Quality assessment and data retrieval

The Newcastle-Ottawa Scale was used for assessing the quality and risk of bias of the selected studies based on inclusion and exclusion criteria. Only papers that passed the quality and risk of bias assessment were included. Data from each original study included was independently extracted and checked in a cross-over manner by two independent researchers. For each study the information retrieved included: study design, population characteristics (sample size of study groups, age, and body mass index), intervention - ovarian stimulation protocol used, and IVF outcomes. The information was then summarized in tables and complemented by a narrative description whenever required due to data heterogeneity.

## Results

### Study selection

The initial search retrieved 664 papers: 402 at Web of Science and 262 at PubMed. After eliminating duplicates, 463 papers were identified for analysis. Among those, 231 papers were excluded based on the information provided in the abstract and 189 additional papers were excluded when full text analysis was considered. Then the remaining 43 papers that met the eligibility criteria underwent risk of bias assessment, which resulted in the exclusion of an additional paper. This yielded a final selection of 42 papers to be included in the systematic review. The flowchart of the search and selection process for studies included in the review is outlined in Fig. 1.

Data retrieved from papers included in this systematic review, revealed that the FF composition of women with PCOS compared to those without PCOS predominantly diverged at the following molecular classes: oxidative stress, inflammatory biomarkers, growth factors and hormones.

### Markers of oxidative stress

Oxidative stress markers assessment in the FF of women with PCOS have been addressed by several studies (Table 1). A lower total antioxidant capacity (TAC) [21–23], along with higher levels of reactive oxygen species (ROS) [22] and 8-Isoprostane (8-IP) [24] has been described in the FF pertaining to women with PCOS comparing to ovulatory non-PCOS women. ROS present chemical properties that often confer reactivity to different biological targets, such as the lipids in membranes, amino acids in proteins, and nucleic acid bases in DNA. Levels of malondialdehyde (MDA), an end-product of lipid peroxidation chain reaction, were also found to be higher in the FF of women with PCOS [21–23].

The activities of several antioxidant enzymes and other molecules involved in redox reaction were also reported to be different in the FF of women with PCOS as compared to controls. Namely, a lower superoxide dismutase (SOD) activity has been described in the FF of PCOS women [23, 25]. In addition, Naigaonkar et al. found that glutathione status was compromised in those patients, as evidenced by decreased levels of reduced glutathione and increased levels of oxidized glutathione [23]. Furthermore, in the same study, the expression of genes involved in antioxidant-related machinery in GCs revealed lower transcript mRNA levels of SOD, glutathione peroxidase (GPx) and glutathione reductase in women with PCOS, in agreement with the activities of the respective enzymes found in the FF [23].

During oxidative stress conditions, oxidizing molecules tend to react with thiol groups (-SH) to form reversible disulphide bonds. Therefore, thiol/disulphide homeostasis can be used as a surrogate marker of oxidative stress. Several authors demonstrated the presence of decreased thiol groups [21, 23, 26], and increased disulphide as well as disulphide/total thiol groups ratio in the FF of women with PCOS [26]. Interestingly, Tola and colleagues also found a positive correlation between FF thiol levels and fertilization rate among women with PCOS [26].

### Inflammation markers

Numerous studies have shown the presence of increased levels of inflammatory markers in the FF of women with PCOS (Table 2). Higher levels of C-reactive protein (CRP) [27, 28] and pro-inflammatory cytokines, including interleukin 6 (IL-6) [21, 27], interleukin 8 (IL-8) [21], interleukin 18 (IL-18) [29], and tumour necrosis factor (TNF-α) [21, 27, 30, 31] were found in the FF of women with PCOS when compared to ovulatory non-PCOS.

Leukaemia inhibitory factor (LIF) is a pleiotropic cytokine and acts as a local regulatory factor in ovary [32]. LIF concentrations in serum and FF of PCOS women were revealed to be lower when compared to controls without PCOS, and negatively correlated with oestradiol concentrations [33]. Progranulin (PGRN) levels, an adipokine recently shown to be associated with low-grade chronic inflammatory state and with a functional role in obesity-related insulin resistance, were reported to be higher in the FF of women with PCOS. PGRN mRNA expression in GCs was found to be higher in PCOS women than in ovulatory non-PCOS, in addition to being higher in overweight when compared to normal-weight women in both PCOS and control patients. Furthermore, PGRN expression levels in the overweight PCOS group were significantly higher than in the overweight control groups [31].

The soluble receptor for advanced glycation endproducts (sRAGE) has been proposed as biomarker of severity in inflammatory and metabolic conditions [34]. Several studies reported the presence of lower sRAGE levels in the FF of women with PCOS when compared to non-PCOS [27, 35, 36]. Furthermore, a multi-adjusted regression analysis showed that higher sRAGE levels in FF predicted the need for lower gonadotrophin doses, higher number of oocytes retrieved and better IVF outcomes in the non-PCOS group [36]. Granulocyte colony-stimulating factor (G-CSF) is a cytokine that stimulates granulocyte cells proliferation and differentiation. Kahyaoglu et al. found that women with PCOS had increased G-CSF levels in both serum and FF compared with ovularory non-PCOS women. The authors hypothesized that as women with PCOS presented more follicles, G-CSF levels could be derived from granulosa cells [37].

Oxidative stress is considered to play an important role in the inflammatory response. Indeed, Artimani and colleagues found a positive correlation between pro-inflammatory cytokines and MDA as well as total oxidant status levels, further corroborating the association between ROS and inflammatory response [21].

### Growth factors

The FF of women with PCOS was found to depict a distinctive growth factor profile, which could potentially contribute to some of the clinical manifestations observed in this condition (Table 3). Namely, elevated levels of vascular endothelial growth factor (VEGF) have been described in the FF of women with PCOS [27, 36, 38]. Further, Savchev and colleagues investigated two isoforms of VEGF present in FF, more specifically VEGF_121_ and VEGF_165_, and found an association between higher VEGF_165_ levels and the diagnosis of PCOS in women with BMI≥ 30 kg/m^2^, and age ≥40 years [39]. The levels of placental growth factor (PlGF), a VEGF family member, were also found to be increased in the FF of women with PCOS when compared to controls. The last study also found that FF of women with PCOS presented lower levels of the soluble form of VEGF receptor 1 (sFlt-1), which is known to bind VEGF and PlGF and block its angiogenic effects on VEGFR [40]. Fang et al. disclosed that the levels of growth differentiation factor-8 (GDF-8) in FF at oocyte retrieval were higher in women with PCOS as compared to the FF of women without PCOS, this study also provided evidence that aberrant expression of GDF-8 in the FF of women with PCOS was associated with subnormal progesterone secretion and poor pregnancy outcomes [41]. Recent animal studies suggested that hepatocyte growth factor (HGF)- and c-Met-mediated epithelial mesenchymal mechanisms are crucial for follicle development [42, 43]. A study conducted by Sahin and co-workers found that the levels of HGF in serum and FF and the mRNA expression of c-Met in GCs were slightly higher in patients with PCOS than in control patients, however these differences were not statistically significant [44]. The fibroblast growth factor (FGF) family is known for being involved in the regulation of ovarian function and follicular development [45]. Liu et al. found that the prevalence of elevated FF testosterone levels was significantly higher in the PCOS patients with elevated FF-FGF13 levels than in those without. Furthermore FF testosterone and increased ovarian volume (> 10 mL for one or both ovaries) were positively correlated with FF-FGF13 in PCOS patients [46].

### Hormones

PCOS is predominantly associated with gonadotrophic axis dysfunction, besides several other hormone imbalances. Elevated levels of LH [49–52], testosterone [53] and Anti-Müllerian hormone (AMH), are the hallmark of gonadotrophic axis dysregulation associated with PCOS [54, 55] (Table 4). AMH is expressed by GCs of pre-antral and small antral follicles. Women with PCOS tend to depict raised AMH circulating levels in parallel to the number of antral follicles [56]. Indeed, several independent studies documented the higher AMH levels in the FF of women with PCOS when compared with controls [50, 52, 57, 58]. Although, the same finding was not observed by Yilmaz et al. in women with PCOS but without obesity or hyperandrogenism [59].

Gonadotrophin and ovulation are regulated by the KISS1/KISS1R system that thus, is essential for female reproductive system [60]. Interestingly, Hu and colleagues found that KISS1 expression levels were significantly upregulated in human granulosa lutein cells obtained from women with PCOS and were highly correlated with AMH serum levels [50].

Women with PCOS often present glucose and energy homeostasis disruptions, along with obesity and metabolic syndrome, which arise associated with raised circulating levels of leptin and insulin [61]. Since leptin is an adipocyte derived hormone, leptin levels are usually proportional to body fat mass [62]. Li and colleagues reported that leptin levels in the FF of infertile women with PCOS was higher when compared to those of women without PCOS. However, this study included normal weight and overweight women (range 19.81–30.62 Kg/m^2^) in both experimental groups [51]. Contrarily, Garruti et al. found that serum and FF leptin levels of non-overweight women with PCOS were lower when compared to those of non-overweight control women [49]. Regarding other adipokines, adiponectin levels were reported to be lower in the FF of lean women with PCOS in comparison with lean non-PCOS women [63]. Furthermore, another study demonstrated that overweight and PCOS women, exhibit increased serum and FF irisin levels compared to normal weight women. Irisin levels were positively correlated with BMI, dyslipidemia and the number of oocytes retrieved and fertilized [64].

PCOS has been shown to be correlated with the endogenous opioid system activity, which could contribute to the pathophysiology of the disorder. Particularly, β-endorphins are opioids involved in female reproductive functions, playing a role in regulating the normal menstrual cycle and possibly in the beginning of puberty [65, 66]. A study conducted by Zhang et al. found higher levels of β-endorphins in the FF of women with PCOS [52], while other authors reported no differences in β-endorphin levels between in FF of women with or without PCOS [67].

Previous studies have reported the association between PCOS and specific thyroid diseases [68]. Gao et al. found higher levels of thyroid-stimulating hormone (TSH) in FF of PCOS women than those in the non-PCOS patients. Furthermore, the expression of TSH receptor in ovarian GCs was significantly upregulated at both mRNA and protein levels in the PCOS group compared with non-PCOS patients [69]. In PCOS patients, TSH levels, both in serum and FF, were negatively correlated with IVF oocyte maturation rate and fertilization rate [69].

### Other molecules

A large amount of data reveals that women with PCOS have an altered FF profile with variations in several biomolecules (Table 5). MicroRNA are small noncoding single-stranded RNA molecules that play important roles in regulating gene expression at the post-transcriptional level. The study of the expression of small non-coding microRNA in FF of women with and without PCOS who underwent the same IVF protocol for subfertility, found 29 miRNAs that differed significantly between the two groups, being the top 7 of these correlated with age, FAI, inflammation and AMH in women with PCOS [73].

Circulating nutrients and exogenous substances can flow through the basal lamina and enter the follicular antrum. A study conducted by Sun and colleagues showed that FF trace elements profile was different in women with PCOS (namely, Cu, Mg, Ca, Ti, As), being the Copper (Cu) concentrations significantly higher. Also, FF Cu levels were positively correlated with FF progesterone and testosterone levels in women with PCOS [74].

Fatty acids can be significant biological markers of aberrant lipid metabolism and have a strong impact on gene expression, which in turn can lead to an altered metabolism, cell growth and differentiation [75]. Niu et al. demonstrated that the metabolic profiles of both plasma and FF indicated higher levels of two long-chain fatty acids, palmitic acid and oleic acid, in women with PCOS compared to controls [76]. Interestingly, the embryo fragmentation score was significantly positively correlated with the oleic acid concentration in all women with PCOS, which may contribute to the mechanisms driving to poor pregnancy outcomes in those patients [76].

FF is known for providing a unique environment for oocyte development and this biological fluid is full of metabolites built up during this process. Therefore, FF metabolites can potentially reflect the oocyte maturation process as well as affect the quality of the mature oocyte. Zhang et al. investigated the FF metabolomics profile from women with PCOS and healthy controls using proton nuclear magnetic resonance (^1^H-NMR). This study revealed that the FF from women with PCOS presented higher levels of glycoproteins, acetate, and cholesterol and lower levels of lactic acid, glutamine, pyruvate, and alanine. The authors hypothesised that differential metabolite profile suggested the presence of altered pyruvate, amino acid and lipid metabolism [77].

Secreted frizzle-related protein-5 (Sfrp-5), a member of the SFRP family, is a novel antagonistic of the Wnt signaling pathway. Recent studies found that Sfrp-5 regulated lipid metabolism, negatively regulated adipogenesis and reduced metabolic dysfunction. Thus, this protein seems to be involved in the pathogenesis of a variety of metabolic diseases [78–80]. Inal and colleagues investigate the effect of serum and FF Sfrp-5 levels on IVF–ICSI outcomes in patients with PCOS and revealed higher FF Sfrp-5 levels in a selected population of nonobese, nonhyperandrogenic PCOS patients. The authors suggest that the Wnt signaling pathway and Sfrp-5 may play an important role in the etiopathogenesis of PCOS [81].

It has been reported that proteoglycans degradation by a disintegrin-like and metalloproteinase with thrombospondin type motifs-1 (ADAMTS-1) is important during folliculogenesis, ovulation, and fertilization via extracellular matrix remodelling [82]. A study conducted by Tola et al. investigated FF aggrecan (proteoglycan) and ADAMTS-1 levels in developing and preovulatory follicles obtained from PCOS and normal ovulatory infertile patients undergoing IVF procedures. The authors found elevated aggrecan and ADAMTS-1 levels in the FF of PCOS women compared with control groups. Also, it was found a positive predictor effect of ADAMTS-1 on implantation, which may indicate that follicular ADAMTS-1 levels could be a potential marker of high-quality embryos for transfer in PCOS patients [83].

Serum amyloid-associated protein has recently been proposed as an inflammatory marker that may be more specific to inflammation related to PCOS. Timur et al. investigated serum and FF amyloid A protein levels in non-obese non-hyperandrogenic patients with PCOS undergoing in IVF [84]. However, no significant difference was found between two groups regarding the serum and FF amyloid A protein levels on the day of oocyte retrieval.

## Discussion

PCOS is a multifactorial disorder characterized by hyperandrogenemia, anovulation, and metabolic dysfunction. Several neural, immune and endocrine axis components seem to be involved in the mechanisms leading to PCOS. The increased pulse amplitude of gonadotropin-releasing hormone release in the hypothalamus promotes LH secretion over FSH, which consequently leads to abnormal sex steroids production and ovarian dysfunction [89]. In the ovary, the hyperandrogenic environment thwarts normal follicular growth, maturation, and ovulation. Besides androgens, circulating AMH levels also tend to be higher in women with PCOS [57, 58]. AMH is a glycoprotein hormone and a member of the transforming growth factor β family of growth and differentiation factors [90]. AMH play a crucial role in male sexual differentiation since is responsible for regression of the female Müllerian ducts. However, AMH has been shown to be involved in postnatal ovarian function whereas this hormone is expressed by GCs of growing follicles from the primary up to the small antral stage [91]. The AMH levels reflect the number of growing follicles recruited from the primordial follicle pool and therefore has been often suggested as a marker for ovarian reserve. Accordingly, Chen and colleagues found a positive correlation between FF AMH levels and antral follicle count in women with PCOS, suggesting that AMH could also be used as a reliable biomarker of ovarian reserve in these patients [58]. Yilmaz and colleagues investigate the FF AMH levels of non-obese non-hyperandrogenemic PCOS, excluding the possible effect of hyperandrogenemia on AMH levels. They observed that FF AMH levels of PCOS patients were higher than the control group although the difference was not significant. Those findings suggest a possible association between FF AMH and androgens [59].

Some of the typical clinical features associated with PCOS include insulin resistance, dyslipidaemia, and abnormal glucose metabolism. Furthermore, the prevalence of overweight and obesity is also higher in women with PCOS, as compared to unaffected women, which tends to exacerbate the symptoms, namely hyperandrogenism and anovulation [92]. Adipokines are adipocyte secreted molecules that have been hypothesized to play a role in PCOS. Adipokine levels including leptin, adiponectin and chemerin were identified to be altered in women with PCOS [28, 49, 51, 63]. Leptin is a peptide hormone, usually released in proportion to adipose tissue mass, that is involved in the control of body weight and energy homeostasis. High leptin levels are frequently found in women with PCOS and suggested to be involved in the pathogenesis of PCOS [51], although are still controversial since other studies present conflicting results [49]. Li and colleagues aimed to investigate whether serum and FF leptin levels, as well as the expression of leptin receptors (Ob-R) and b-R signaling in GCs were altered in women with PCOS. The authors reported higher leptin levels in serum and FF and decreased expression of p-STAT3 in GCs of women with PCOS compared to non-PCOS women. Further analysis showed that those findings were related to poor IVF outcome, suggesting that abnormalities of leptin secretion and signaling pathway could be involved in PCOS-associated infertility [51]. On the other hand, Garruti and colleagues reported lower serum and FF leptin levels in non-overweight women with PCOS (22.55±2.6 Kg/m^2^) when compared to BMI-matched (22.55±2.6 Kg/m^2^) non-overweight non-PCOS women. The disparities between studies could be attributed to demographic differences of study cohorts (ethnicity, age, BMI) and/or heterogeneity of the syndrome itself.

Oxidative stress and chronic low-grade inflammatory status have been anticipated as key contributors to PCOS pathogenesis since several reports have disclosed higher oxidative stress and inflammatory mediators in those women. ROS can induce inflammatory response prompting the release of cytokines, and both phenomena are directly associated with PCOS comorbidities including obesity, insulin resistance, dyslipidemia and hyperandrogenism. As a matter of fact, a study conducted by Artimani and colleagues demonstrated that in women with PCOS there was a positive correlation between inflammatory cytokines and pro-oxidant agents and a negative correlation with antioxidant levels [6]. This is very relevant since the physiological balance between pro-oxidants and antioxidants is deemed to ensure several reproductive processes including ovarian steroid genesis, oocyte maturation and ovulation [93]. However, an altered redox homeostasis is often observed in PCOS. Indeed, several studies included in this review demonstrated a tendency for women with PCOS to have higher levels of pro-oxidant markers (e.g. ROS/TOS and MDA) [6, 22, 23] and lower antioxidant capacity (e.g. TAC, free thiols groups and SOD activity)[6, 22, 23, 25]. Lipid peroxidation (LPO) is a well characterized consequence of oxidative stress on cell membrane structure and function. LPO is a process characterized by the molecular modification of lipids containing carbon-carbon double bond(s), especially polyunsaturated fatty acids, when exposed to free radicals [94]. During LPO different aldehydes can be formed as secondary products, among which malondialdehyde (MDA) and 4-hydroxynonenal (4-HNE) have been widely used as OS surrogate markers. Women with PCOS have been reported to present higher MDA levels than non-PCOS patients [6, 22, 23]. Naigaonkar et al. also found that women with PCOS and insulin resistance had higher levels of LPO and hypothesized that those levels could be a consequence of free fatty acid levels abundancy and accumulation in the mitochondria, which is a prominent source of ROS [23]. The human body holds a complex antioxidant defense system that relies on endogenous enzymatic and non-enzymatic antioxidants, which are responsible to scavenge oxidants and prevent oxidative stress-related damages. The three major antioxidant enzymes include SOD, catalase (CAT) and glutathione peroxidase (GPx) [95]. The metalloenzyme SOD promotes the dismutation of superoxide (O_2_•−) into molecular oxygen (O_2_) and hydrogen peroxide (H_2_O_2_) and is considered the first line of defense against ROS. There is also evidence that the FF of women with PCOS has lower SOD activity and GCs have lower SOD transcript expression [23, 25]. These findings demonstrated that redox homeostasis in follicular microenvironment of women with PCOS is altered towards a pro-oxidant environment that can compromise oocyte quality. Furthermore, GCs seem to play a critical role in maintaining redox balance within the follicle, as transcript expression of antioxidant enzymes in GCs was in line with its activity at the FF [23]. Thiol redox reaction is another mechanism of protection against OS. Thiol is a compound that contains a sulfhydryl group (-SH) functional group. The –SH group in thiols reacts with oxidants to form disulphide bonds, exerting a protective effect against OS. Consistent with the aforementioned, disulphide levels were found to be higher [26] while thiol levels were reported as decreased [23, 26] in the FF of women with PCOS than without PCOS. Further, a positive correlation between fertilization rate and thiol levels among women with PCOS was also reported [26].

Oxidative stress and inflammation are tightly linked pathophysiological processes. The role of inflammation in oocyte quality and reproductive function has been broadly investigated. TNF-α and IL-6 are proinflammatory cytokines involved in modulation of inflammatory response. These cytokines have been associated with obesity, insulin resistance and attendant comorbidities, such as type 2 diabetes [96]. Indeed, FF levels of TNF-α and IL-6 were found to be higher in infertile women with PCOS compared to control women [6, 27, 31]. Some authors even postulated that it is the imbalance between pro- and anti-inflammatory cytokines that leads to altered steroidogenesis, delayed follicular maturation and ovarian dysfunction, which are some of PCOS key clinical features [97]. RAGE is a transmembrane protein that belongs to the immunoglobulin superfamily. This receptor binds a variety of stress associated molecules, including advanced glycation end products (AGEs), a family of compounds formed in the presence of glucose by non-enzymatic alteration of proteins, lipids, and nucleic acids [98]. The sRAGE is an extracellular form of RAGE that bind its ligands (AGEs), thus interrupting the adverse intracellular signaling triggered by the AGE-RAGE axis [99]. There is growing evidence supporting the AGE-RAGE system contribution to increased oxidative stress and inflammation [100]. This system has been implicated in the pathogenesis of multiple metabolic diseases and more recently in PCOS and infertility [99]. It has been demonstrated that women with PCOS have lower FF sRAGE levels when compared to non-PCOS, however the effects of reduced sRAGE levels in PCOS remains unclear [27, 35, 36]. AGE–RAGE interactions can also stimulate VEGF production and consequently modify the follicular environment [99]. A study conducted by Wang and colleagues demonstrated that treatment of GCs, isolated from women with PCOS, with recombinant sRAGE decreased VEGF mRNA expression [36]. VEGF is a potent angiogenic factor that was first described as an essential growth factor for vascular endothelial cells. Macrophages and GCs are considered the most significant sources of VEGF in female reproductive tract. VEGF expression was found to be associated to important events in the course of the ovarian cycle, including follicular growth, ovulation, corpus luteum development, and ovarian steroidogenesis [101]. VEGF has been suggested to play a role in the selection of the dominant follicle by increasing vascularization and, VEGF seems to be involved in the ovulation process through the activation of plasminogen activator system and plasminogen activator inhibitor 1 [102]. Numerous studies found higher VEGF levels in the FF of women with PCOS compared to control women [27, 36, 38, 39]. It was proposed that the elevated circulating VEGF levels in women with PCOS may partially explain the ovarian stroma hypervascularization, which characterizes PCOS [103]. In fact, the profuse vascularization has been hypothesized to facilitate abnormal theca interna cell growth, responsible for androgen steroidogenesis. Kudsy et al. also hypothesized that granulosa lutein cells VEGF secretion could be stimulated by insulin, since GCs of women with PCOS are more sensitive to insulin [38]. Since insulin resistance tends to be more frequent in women with PCOS than in the general population, it is reasonable to hypothesize that the consequent hyperinsulinism could contribute for VEGF hypersecretion [104]. Given the aforementioned, VEGF appears to play a critical role in the pathophysiology of PCOS.

Recently, approaches in “omics” technologies have been explored in an attempt to further understand this heterogeneous and complex disorder. Amongst these, metabolomics, which examines the fingerprints of all metabolites in a biological system, allows the investigation of metabolite pathways that may provide new insights into the underlying biology of PCOS. Zhang and colleagues using ^1^H NMR metabolomics approach explored the metabolic variance at the FF from women with PCOS and healthy controls [77]. A total of 9 metabolite regions were shown to differentiate the FF from women with PCOS and healthy women. The decreased levels of pyruvate and lactate and increased levels of acetate suggested the occurrence of shifts in pyruvate metabolism and glycolysis. Aberrant insulin level may underlie these changes, as it contributes to decreased glucose uptake and thus glycolytic flux [77]. Also, it is possible that the decreased levels of some amino acids, namely alanine and glutamine, could be attributed to a compensatory increase in amino acids consumption as cellular substrate to produce energy. The increased glycerol and lipid levels, namely cholesterol is not unexpected since these are typical signs of dyslipidemia, which is a common comorbidity associated with PCOS. It is suggested that impaired lipase expression and altered lipolysis caused by insulin resistance, may be responsible for those events [105]. Glycoproteins are closely related to several inflammatory disorders and the high glycoprotein level found in the FF of women with PCOS suggest that the oocytes’ environment might also be characterized by low-grade inflammation [106]. Interestingly, the authors reported negative correlations between embryos quality and several amino acids, including creatine, leucine, isoleucine, as well as tyrosine. Most particularly, a negative correlation between glucose and both 2PN fertilization rate and cleavage rate and positive correlations between acetoacetate and 3-hydroxy-butyrate and 2PN fertilization rate have been reported in women with PCOS. The latest observations suggest that the metabolic shifts occurring in GCs and detected in the FF of women with PCOS might affect the ART process.

**Fig. 2:**
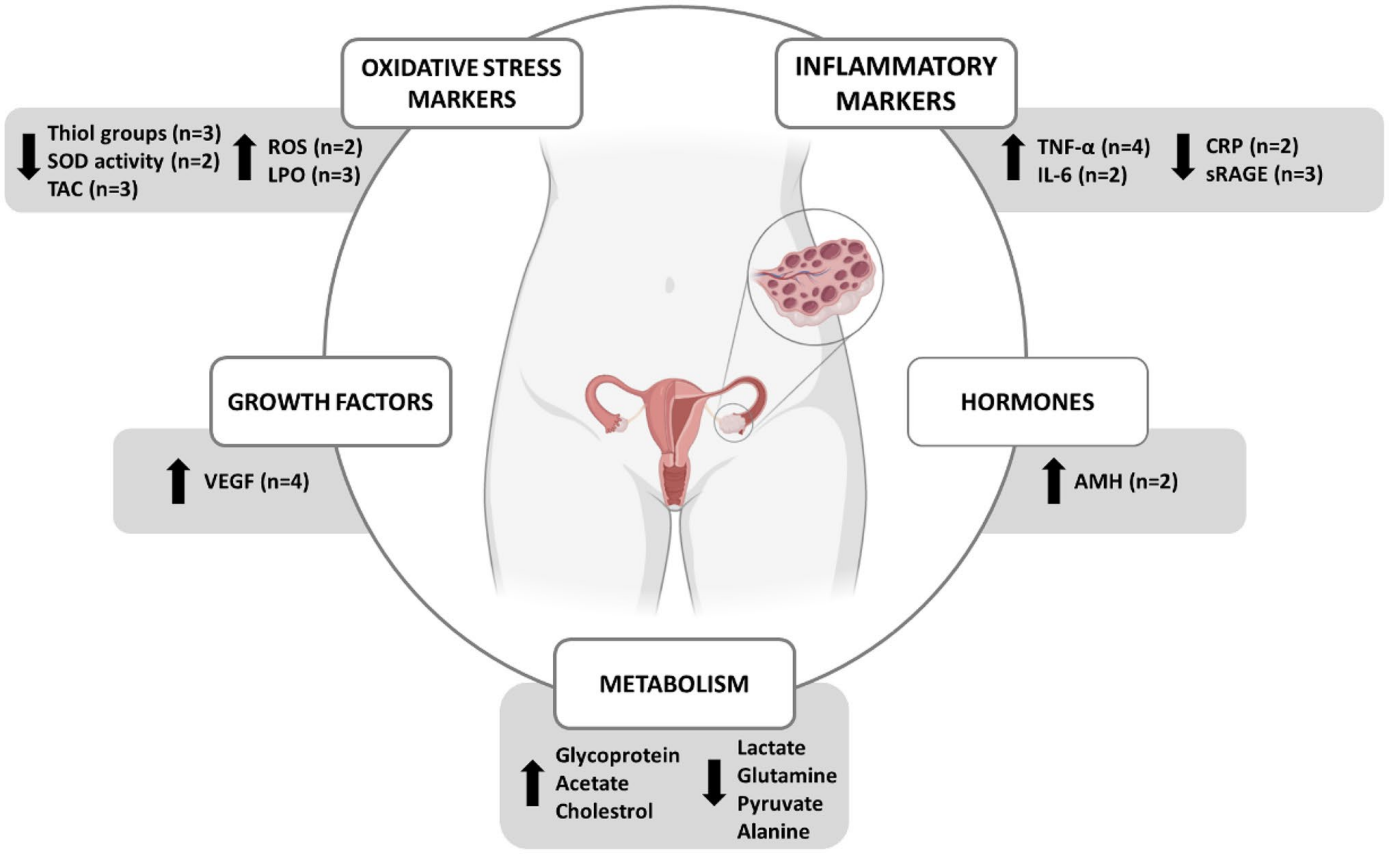
Main changes on the composition of follicular fluid in women with polycystic ovary syndrome. Abbreviations: AMH - anti-mullerian hormone; CRP – c-reactive protein; IL-6 – interleukin 6; LPO – lipid peroxidation; ROS – reactive oxygen species; SOD – superoxide dismutase; sRAGE – soluble receptor for advanced glycation end products; TAC – total antioxidant capacity; TNF-α – tumor necrosis factor; VEGF - vascular endothelial growth factor

Taken all together, the data retrieved from papers included in this review revealed that the FF composition of women with PCOS compared to those without PCOS predominantly differ on the levels of oxidative stress and inflammatory biomarkers, growth factors, hormones and metabolites (Fig. 2). The identification of the metabolic pathways and molecular mechanisms involved in PCOS pathophysiology might allow the identification of specific biomarkers, which in turn could potentially be useful for early diagnosis of this endocrinopathy and be the outset for future tailored pharmacological therapies.

## Conclusion

The characterization of FF and its molecular profile opens a window of opportunity to further understand PCOS. The FF is a biological matrix that is in intimate contact with the oocyte and contains a variety of bioactive molecules, which contribute for follicle development and maturation. The identification of molecular shifts within the FF harbors the potential of understanding the pathways involved in the ovarian dysfunction observed in women with PCOS. In conclusion FF can be a promising tool to identify novel biomarkers for the PCOS diagnosis as well to the development of targeted therapeutics for the treatment of PCOS.

**Table 1:** Summary of studies comparing oxidative stress markers in follicular fluid of PCOS and non-PCOS women

**First Author** **Publication Year**	**Experimental Design**	**Population**			**IVF/ICSI Treatment** **Ovarian Induction**	**Outcome Measures**	**Results**
		**Groups**	**Age (years)**	**BMI (kg/m**^**2**^**)**			
**Oxidative stress markers**							
Chattopadhayay, R. 2010 [22]	Prospective cohort	PCOS (n=35) Ovulatory non-PCOS (n=32)	29.9 ± 2.0 29.9 ± 2.2	23.3 ± 1.2 23.0 ± 1.2	ICSI GnRH agonist, rFSH	**FF:** ROS, LPO and TAC **Serum:** TSH, PRL **ICSI outcomes:** MII oocytes (n), FR (%), Pregnancy rate (%), Miscarriage rate (%), Grade I + II embryo formation (%), Meiotic spindle present (%)	**PCOS:** ↑ROS, LPO ↓TAC FF ROS and LPO levels were higher* and TAC levels lower* in oocytes without meiotic spindle (vs. oocytes with meiotic spindle) from both PCOS and control groups. Fertilization rate and the number of embryos were lower* in oocytes without meiotic spindle (vs. oocytes with meiotic spindle) among women with PCOS.
Seleem, A. K. 2014 [25]	Case-control	PCOS (n=20) Ovulatory non-PCOS (n=20)	31.2 ± 1.1 29.4 ± 2.1	31.3 ± 3.2 29.2 ± 3.6	ICSI GnRH agonist, rFSH	**FF:** SOD **Serum:** SOD **ICSI outcomes:** FR (%), Embryo quality (n), Transferred embryo (n), Pregnancy (n)	**PCOS:** ↓SOD The mean Cu/Zn-SOD mRNA expression in FF cells was lower* in women with PCOS. FF SOD activity and SOD mRNA expression were not significantly between fertilized and non-fertilized oocytes in both groups. SOD activity in FF had no effects on fertilization rate or embryo quality.
Yilmaz, 2016 [9]	Prospective cross-sectional	PCOS (n=22) Ovulatory non-PCOS (n=41)	30.0 (25-34) 32.0 (25-34)	24.7 ± 2.9 23.8 ± 1.4	IVF GnRH agonist protocol, rFSH	**FF:** TAC **IVF outcomes:** peak E_2_, endometrial thickness, retrieved oocytes (n), MII oocytes (n), FR (%), clinical pregnancy rate (%)	FF TAC levels, fertilization and clinical pregnancy rates were not significantly different between groups. N. of MII oocytes were higher* in women with PCOS. FF TAC was positively correlated* with clinical pregnancy rate in the whole group.
Tola, E. N. 2018 [26]	Cross-sectional	PCOS (n=22) Ovulatory non-PCOS (n=20)	28.5 ± 3.6 30.6 ± 4.4	27.9 ± 5.9 25.9 ± 4.4	ICSI GnRH antagonist, rFSH	**FF:** thiol/disulphide parameters **IVF outcomes:** Retrieved oocytes (n), GV/MI/MII oocytes (n), FR (%), Embryo (n)	**PCOS:** ↑disulphide levels, disulphide/native thiol and disulphide/total thiol ratios ↓native thiol levels and native thiol/total thiol ratio FR among women with PCOS was positively correlated with native thiol levels and total thiol in FF. Native thiol levels are positive predictors of FR in PCOS.
Gongadashetti, K. 2021 [24]	Cross-sectional	PCOS (n=43) Ovulatory non-PCOS (n=57)	30.5 ± 3.8 (22-39) 32.2 ± 3.8 (23-40)	25.3 ± 4.1 25.8 ± 3.7	IVF GnRH antagonist	**FF:** ROS, TAC and 8-IP **IVF outcomes:** Oocytes retrieved (n), Oocytes fertilized (n), Cleavage rate (%), Embryos (n) Grade I, II, III, IV embryos (n), Pregnancy (n), Miscarriage (n)	**PCOS:** ↑8-IP The 8-IP levels were higher* in women with PCOS who had a miscarriage.
Naigaonkar, A. 2021 [23]	Cohort	PCOS (n=50) Ovulatory non-PCOS (n=71)	31.0 (27.0–33.0) 28.0 (26.5–31.0)	25.7 (22.68–29.87) 24.7 (22.5–26.5)	IVF GnRH agonist, hMG	**FF:** SOD, CAT, PON1, GR, GSH, GSSG, GPx, TAC, AOPP, LPO, total thiol levels, SHBG, E_2_, P_4,_ TT, and fasting insulin **Serum:** SHBG, E_2_, P_4,_ TT, fasting glucose, fasting insulin. **IVF outcomes:** Follicles (n), Oocytes (n), MII oocyte (n, %), Fertilized MII oocyte (n), Grade I embryos (%), FR (%), Pregnancy rate per embryo transfer (%)	**PCOS:** ↑TT, FAI, LPO, GSSG, AOPP ↓P_4_, SHBG, SOD, PON1, GSH, GPx, GR, total thiols, TAC Transcript levels of SOD, GPX, GR, and GCL in GCs were lower* in women with PCOS. PON1 was positively correlated with pregnancy outcome, and total glutathione with % grade I embryos and pregnancy outcome in control group. PON1 activity showed a positive correlation with n. of fertilized oocytes and % grade I embryos, in women with PCOS.

*AOPP* advanced oxidation protein products, *BMI* body mass index, *CAT* catalase, *Cu/Zn-SOD* copper, zinc superoxide dismutase, *E*_*2*_ oestradiol, *FAI* free androgen index, *FF* follicular fluid, *FR* fertilization rate, *GnRH* gonadotropin-releasing hormone, *GPx* glutathione peroxidase, *GR* glutathione reductase, *GSH* glutathione, *GSSG* oxidized glutathione, *GV* germinal vesical, *hMG* human menopausal gonadotropin, *ICSI* intracytoplasmic sperm injection, *8-IP* 8-isoprostane, *IVF in vitro* fertilization, *LPO* lipid peroxidation, *MI* metaphase I, *MII* metaphase II, *mRNA* messenger ribonucleic acid, *n* number, *P*_*4*_ progesterone, *PCOS* polycystic ovarian syndrome, *PON1* paraoxonase 1, *rFSH* recombinant follicle stimulating hormone, ROS reactive oxygen species, SHBG sex hormone-binding globulin, SOD superoxide dismutase, *TT* testosterone, *TAC* total antioxidant capacity, *TSH* thyroid stimulating hormone

**Table 2:** Summary of studies comparing inflammatory markers in follicular fluid of PCOS and non-PCOS women

**First Author** **Publication Year**	**Experimental Design**	**Population**			**Ovarian Induction IVF/ICSI**	**Outcome Measures**	**Results**
		**Groups**	**Age (years)**	**BMI (kg/m**^**2**^**)**			
**Inflammatory markers**							
Kahyaoglu, I. 2015 [37]	Case-control	PCOS (n=22) Ovulatory non-PCOS (n=22)	27.6 ± 0.9 28.3 ± 0.7	26.1 ± 1.3 24.4 ± 2.3	IVF GnRH agonist long protocol, rFSH	**FF:** G-CSF **Serum:** G-CSF **IVF outcomes:** endometrial thickness, retrieved oocytes (n), MII oocytes (n), FR (%), clinical pregnancy rate (%)	**PCOS:** ↑G-CSF Neutrophil count and neutrophil/leukocyte ratio were both significantly higher in women with PCOS than in controls. N. of retrieved and MII oocytes were higher in PCOS group.
Wang, B. 2016 [27]	Prospective cohort	PCOS (n=39) Ovulatory non-PCOS (n=35)	27.6 ± 3.2 28.4 ± 2.9	21.0 ± 2.1 21.0 ± 1.6	IVF GnRH agonist, rFSH	**FF:** TNF-α, IL-6, CRP, VEGF and sRAGE **IVF outcomes:** oocytes retrieved (n), fertilization rate (%), high quality embryos rate (%)	**PCOS:** ↑TNF-α, IL-6, CRP and VEGF ↓sRAGE FF sRAGE was inversely correlated* with gonadotropin dose and total in PCOS. After adjusting for age and Gn dose, FF sRAGE protein levels, inversely related to VEGF, TNF-α, IL-6 and CRP.
Garg, D. 2017 [35]	Prospective cohort	PCOS (n=12) Ovulatory non-PCOS (n=13)	31.5 [29.2-34.5] 35.0 [21.0-37.5]	24.5 [22.5-33.7] 25.0 [21.5-29.0]	IVF/ICSI GnRH antagonist protocol	**FF:** sRAGE, CML, 25-hydroxy-vitamin D **Serum:** AMH, TSH **IVF/ICSI outcomes:** oocytes retrieved (n), MII oocytes (n), fertilized oocytes (n), clinical pregnancy rate (%)	**PCOS:** ↓sRAGE FF sRAGE positively correlated* with 25-hydroxy-vitamin D (in each group and in the whole group). FF sRAGE positively correlated* with CML, in control group.
Niu, Z. 2017 [30]	Cohort	PCOS (n=30) PCOS with MS (n=30) Ovulatory non-PCOS (n=30)	30.2 ± 3.9 30.6 ± 3.5 29.4 ± 3.7	22.6 ± 2.9 32.1 ± 3.6 22.3 ± 2.6	IVF GnRH antagonist	**FF:** TNF-α, IL-1b, IL-2, IL-4, IL-5, IL-6, IL-7, IL-8, IL-10, IL-12, IL-13, IL-17, IL-18, TC, TG, LDL, HDL IFN-γ, MCP-1, G-CSF, and RANTES **Serum:** FSH, LH, E_2_, P_4_, TC, TG, LDL and HDL **IVF outcomes:** 2PN (%), Embryos (%), top-quality embryos (%)	**PCOS MS:** ↑TNF-α The percentage of top-quality embryo was lower* in the women with PCOS and MS than in the other two groups. The FF G-CSF showed a trend toward negative relationship with TG and TC. TNF-α concentration was positively associated with TG.
Wang, B. 2017 [36]	Prospective cohort	PCOS (n=39) Ovulatory non-PCOS (n=35)	27.7 ± 3.3 28.4 ± 3.0	22.1 ± 2.2 21.0 ± 1.7	IVF GnRH agonist protocol, rFSH	**FF:** sRAGE and VEGF **IVF outcomes:** total gonadotropin dose, duration of stimulation, oocytes retrieved (n), fertilization rate (%), high-quality embryos rate (%)	**PCOS:** ↑VEGF ↓sRAGE FF sRAGE levels were positively correlated with the number of oocytes retrieved in the control group. FF sRAGE protein levels in women with PCOS were negatively related to the total dose of Gn. The treatment of human GCs (PCOS) with recombinant sRAGE decreased VEGF and SP1 mRNA and protein expression and pAKT levels in a dose-dependent manner.
Zhou, D. 2017 [31]	Cohort	PCOS (n=39) BMI < 25 (n=24) BMI ≥ 25 (n=15) ovulatory non-PCOS (n=53) BMI < 25 (n=35) BMI ≥ 25 (n=18)	29.7 ± 3.5 31.2 ± 3.8 31.6 ± 4.7 31.2 ± 2.9	22.3 ± 1.4 27.8 ± 1.6 20.4 ± 2.0 26.0 ± 1.3	IVF/ICSI GnRH agonist, rFSH	**FF:** PGRN, TNF-α, IL-6 and MCP-1 **Serum:** E_2_, LH, FSH, TT, fasting blood glucose **IVF/ICSI outcomes:** Retrieved oocytes(n), Oocyte’s Fertilization(n), Grade I/II embryos(n)	**PCOS:** ↑PGRN, TNF-α FF PGRN levels and GCs mRNA expression of PGRN were higher* in women with PCOS than in controls, and higher* in overweight than in the normal-weight women. FF TNF-α levels were higher in women with PCOS than controls; and higher rate of overweight than normal-weight controls. In women with PCOS, FF PGRN was positively correlated* with TT and FF TNF-α, but negatively correlated* with n. of retrieved oocytes.
Artimani, T. 2018 [21]	Cross-sectional	PCOS (n=21) Ovulatory non-PCOS (n=21)	28.8 ± 4.9 28.6 ± 4.7	28.2 ± 5.3 29.2 ± 4.5	ICSI GnRH agonist, rFSH	**FF:** thiol groups TAC, MDA, TOS, TNF-α, IL-6, IL-8, and IL-10 l. **ICSI outcomes:** Oocyte (n), MII oocyte (n), Fertilization rate (%), Embryo (n)	**PCOS:** ↑MDA, TOS, TNF-α, IL-6 and IL-8 ↓TAC, thiol groups and IL-10 TNF-α levels were positively correlated* with MDA and TOS and negatively correlated* with TAC and thiol groups. IL-6 and MDA levels were positively correlated with IL-10 levels and TAC. IL-8 and IL-10 were negatively correlated* with TAC and TOS levels, respectively. The n. of retrieved oocytes was higher* in women with PCOS.
Li, Z. 2018 [33]	Case-control	PCOS (n=40) Ovulatory non-PCOS (n=40)	28.4 ± 3.7 30.2 ± 4.2	26.7 ± 3.0 25.2 ± 2.3	IVF GnRH long agonist, rFSH	**FF:** LIF, E_2_ and P_4_ **Serum:** LIF, E_2,_ FSH, LH, P_4_, TT, and PRL **IVF outcomes:** pregnancy rate (%)	**PCOS:** ↓LIF Serum and FF LIF levels were negatively correlated with E_2_ in women with PCOS. LIF concentrations in embryo culture medium were higher* in women who achieved pregnancy in both groups.
Wang, Y. 2019 [28]	Cohort	PCOS (n=30) Ovulatory non-PCOS (n=23)	27.4 ± 4.0 27.7 ± 3.3	21.2 ± 1.9 20.2 ± 1.6	IVF GnRH antagonist protocol	**FF:** CRP, chemerin FSH, LH, TT, E_2_, P_4_ **Serum:** FSH, LH, TT, E_2_, P_4_, PRL, AMH and TSH **IVF outcomes:** oocytes retrieved (n), oocyte utilization rate and high-quality embryo rate	**PCOS: ↑**LH, TT, CRP, chemerin The mRNA expression of chemerin and its receptors in granulosa cells from women with PCOS were higher* than those from non-PCOS. FF chemerin was positively correlated with TT and LH in FF.
Zhang, H. 2020 [29]	Cohort	PCOS (n=60) Ovulatory non-PCOS (n=60)	29.5 ± 3.9 30.5 ± 3.7	24.5 ± 2.6 22.5 ± 2.3	IVF GnRH antagonist, rFSH and HMG	**FF:** IL-18 and IL-18BP **Serum:** E_2_, TT, LH, FSH, IL-18 and IL-18BP **IVF outcomes:** oocytes retrieved (n), MII oocytes (n), embryos transferred (n), Top-quality embryo (%), fertilization rate (%), Pregnancy rate (%)	**PCOS:** ↑IL-18 ↓IL-18BP FF IL-18 levels were higher* than in serum in women with PCOS. FF IL-18 was higher* in overweight women with PCOS compared to normal weight women with PCOS. FF IL-18 levels in women with PCOS were positively* correlated with TT and negatively correlated with E_2_ levels. In women with PCOS women, FF IL-18 was positively correlated* with the n. of oocytes retrieved, and negatively correlated* with the n. of MII oocytes and top-quality embryos.

*AMH* antimüllerian hormone, *BMI* body mass index, *CML* N-carboxymethyl-lysine, *CRP* C-reactive protein, *E*_*2*_ oestradiol, *FF* follicular fluid, *FR* fertilization rate, *FSH* follicle stimulating hormone, *G-CSF* granulocyte colony-stimulating factor, *GnRH* gonadotropin-releasing hormone, *GCs* granulosa cells, *GV* germinal vesical, *HDL* high-density lipoprotein, *hMG* human menopausal gonadotrophins, *ICSI* intracytoplasmic sperm injection, *IL* interleukin, *IFN-γ* Interferon gamma, *IVF in vitro* fertilization, *LIF* leukemia inhibitory factor, *LH* luteinizing hormone, *LDL* low-density lipoprotein, *MCP-1* Monocyte chemoattractant protein-1, *MDA* malondialdehyde, *MI* metaphase I, *MII* metaphase II, *mRNA* messenger ribonucleic acid, *MS* metabolic syndrome, *n* number, *P*_*4*_ progesterone, *PCOS* polycystic ovarian syndrome, *PGRN* progranulin, *PRL* prolactin, *2PN* two pronuclei (fertilized oocytes), *RANTES* Regulated on activation, normal T expressed and secreted, *rFSH* recombinant follicle stimulating hormone, *SHBG* sex hormone-binding globulin, *sRAGE* soluble receptor for advanced glycation end products, *TAC* total antioxidant capacity, *TNF-α* tumor necrosis factor alpha, *TOS* total oxidant status, *TC* total cholesterol, *TG* triglycerides, *TT* testosterone, *VEGF* vascular endothelial growth factor

**Table 3:** Summary of studies comparing growth factors in follicular fluid of PCOS and non-PCOS women

**First Author** **Publication Year**	**Experimental Design**	**Population**			**Ovarian Stimulation IVF/ICSI**	**Outcome Measures**	**Results**
		**Groups**	**Age (years)**	**BMI (kg/m**^**2**^**)**			
**Growth factors**							
Savchev, S. I. 2010 [39]	Prospective cohort study	PCOS (n=6) Ovulatory non-PCOS (n=11)	27.6 (25-31) 26.6 (22-30)	29.5 (17-51) 22.0 (19-29)	ICSI GnRH agonist or antagonist protocols, rFSH	**FF:** VEGF_121_, VEGF_165_ and sFlt-1 **ICSI outcomes:** oocytes retrieved (n), FR (%), embryos transferred (n)	Higher FF VEGF_165_ levels* in women with PCOS, BMI > 30 and age > 40 years. Lower FF VEGF_165_ levels were associated* clinical pregnancy.
Şahin, N. 2013 [47]	Prospective cohort study	PCOS (n=21) Ovulatory non-PCOS (n=38)	29.3 ± 0.9 31.7 ± 0.8	24.5 ± 0.7 24.4 ± 0.5	ICSI GnRH agonist protocol, rFSH	**FF:** HGF **Serum:** FSH, LH, P_4_, E_2_, TT, DHEAS, A **ICSI Outcomes:** total FSH dose, oocytes retrieved (n), MII oocytes (n), FR (%), clinical pregnancy rate (%)	No significant differences were found in serum and FF HGF levels between groups. Higher c-Met expression* in GCs of fertilized oocytes (vs. non-fertilized oocytes) Higher FF HGF levels* in the grade 1 embryos (vs. grades 2-4 embryos).
Tal, R. 2014 [40]	Prospective cohort	PCOS (n=14) Ovulatory non-PCOS (n=14)	30.1 ± 4.4 30.8 ± 3.7	25.5 ± 5.6 24.9 ± 3.9	IVF/ICSI GnRH agonist (n=8 for each group) or GnRH antagonist (n=6 for each group) protocols, combination of rFSH and hMG	**FF:** PIGF and sFlt-1 **Serum:** AMH, PIGF and sFlt-1 **IVF/ICSI outcomes:** oocytes retrieved (n), FR (%), pregnancy rate (%)	**PCOS:** ↑FF PIGF ↓FF sFlt-1 FF PIGF was positively correlated* with n. of oocytes retrieved and serum AMH and negatively correlated* with age. PlGF bioavailability (PlGF/sFlt-1 ratio) in FF was greater in PCOS women compared with non-PCOS controls.
Kudsy, M. 2016 [38]	Prospective cross-sectional	PCOS (n=40) Ovulatory non-PCOS (n=40)	28.6 ± 4.3 28.4 ± 5.9	27.2 ± 5.7 26.1 ± 4.3	IVF/ICSI GnRH long agonist protocol, rFSH or hMG	**FF:** VEGF **Serum:** VEGF **IVF/ICSI outcomes:** retrieved oocyte (n), MII oocyte (n and %), fertilized oocyte (n), embryos (n), FR (%)	**PCOS:** ↑VEGF FF VEGF levels were lower* in women who achieved pregnancy vs. non-pregnant both in PCOS and control.
Liu, Y. 2018 [48]	Cross-sectional	PCOS (n=43) Ovulatory non-PCOS (n=32)	28 (23.0-33.0) 27 (25.0-30.0)	22.6 (20.2-24.0) 20.3 (19.0-23.5)	IVF/ICSI GnRH antagonist protocol	**FF:** FGF13 and FGF21, IL-6, TT, E_2_, P_4_, LH, FSH and SHBG **IVF/ICSI outcomes:** oocytes retrieved (n), MII oocytes rate (%), FR (%), high-quality embryo rate (%)	FF FGF13 levels were positively correlated with FF TT levels in women with PCOS. FF FGF13 levels in women with PCOS was negatively associated* with MII oocyte rate.
Fang, L. 2020 [41]	Prospective cohort	PCOS (n=40) Ovulatory non-PCOS (n=40)	29.3 ± 0.6 29.3 ± 0.6	24.4 ± 0.5 22.2 ± 0.5	IVF GnRH agonist protocol, rFSH	**FF:** GDF-8 **Serum:** FSH, LH, E_2_, P_4_, TT, PRL, GDF-8 **IVF outcomes:** oocytes retrieved (n), MII oocytes (n), pregnancy outcomes	**PCOS:** ↑GDF-8 Serum and FF GDF-8 levels were lower* in women with PCOS who achieved pregnancy vs non-pregnant. GDF-8 resulted in greater inhibition of StAR expression in granulosa-lutein cells from women with PCOS than without-PCOS. Low serum P_4_ levels were associated with poor pregnancy outcomes in women with PCOS.

*A* androstenedione, *AMH* antimüllerian hormone, *BMI* body mass index, *c-Met* mesenchymal epithelial transition factor, *DHEAS* dehydroepiandrosterone sulfate, *E*_*2*_ oestradiol, *FF* follicular fluid, *FGF13* fibroblast growth factor 13, *FGF21* fibroblast growth factor 21, *FR* fertilization rate, *FSH* follicle stimulating hormone, *GDF-8* growth differentiation factor 8, *GnRH* gonadotropin-releasing hormone, *HGF* hepatocyte growth factor, *hMG* human menopausal gonadotrophins, *ICSI* intracytoplasmic sperm injection, *IL* interleukin, *IVF in vitro* fertilization, *LH* luteinizing hormone, *MI* metaphase I, *MII* metaphase II, *mRNA* messenger ribonucleic acid, *n* number, *P*_*4*_ progesterone, *PCOS* polycystic ovarian syndrome, *PIGF* placental growth factor, *PRL* prolactin, *rFSH* recombinant follicle stimulating hormone, *sFlt-1* soluble Fms-like tyrosine kinase-1, *SHBG* sex hormone-binding globulin, *StAR* steroidogenic acute regulatory, *TT* testosterone, *VEGF* vascular endothelial growth factor

**Table 4:** Summary of studies comparing hormones in follicular fluid of PCOS and non-PCOS women

**First Author** **Publication Year**	**Experimental Design**	**Population**			**IVF/ICSI Treatment** **Ovarian Induction**	**Outcome Measures**	**Results**
		**Groups**	**Age(years)**	**BMI (kg/m**^**2**^**)**			
**Hormones**							
Li, M. G. 2007 [51]	Case-control	PCOS (n=31) Ovulatory non-PCOS (n=79)	28 (22–35) 29 (23–37)	23.4 (19.8-30.6) 21.4 (16.7-28.5)	IVF GnRH agonist protocol, rFSH	**FF:** leptin **Serum:** LH, FSH, E_2_, P_4_, TT, leptin levels **IVF outcomes:** oocytes retrieved (n), FR (%), good quality embryo (%), implantation rate (%), pregnancy rate (%)	**PCOS:** ↑leptin The p-STAT3 expression was lower* in PCOS compared with control, whereas there were no significant differences in SOCS3 expression in GCs. Serum and FF leptin levels were higher in the failed pregnancy PCOS subgroup vs. successful pregnancy PCOS subgroup.
Nafiye, Y. 2010 [70]	Cross-sectional	PCOS (n=36) non-PCOS with infertile male partners (n=23) non-PCOS women with unexplained infertility (n=38)	29.6 ± 5.1 28.4 ± 6.0 i 29.7 ± 4.4	26.3 ± 2.9 24.8 ± 3.3 24.7 ± 2.9	400 μg/day of preconceptional folic acid IVF GnRH agonist protocol, rFSH	**FF:** glucose, insulin, Homocysteine, TT, A, P_4_, DHEA **Serum:** E_2_, TT, A, P_4_, DHEA, insulin, glucose, and Homocysteine **IVF outcomes:** follicle (n), retrieved and MII oocytes (n), embryo (n), transferred embryo (n), clinical pregnancy rate (%)	Despite elevated serum insulin, HOMA-IR, and homocysteine levels, and their effects on oocyte numbers and maturation in PCOS patients, there were no differences in follicular parameters and clinical pregnancy rates.
Yilmaz, N. 2012 [59]	Cross-sectional	PCOS (n=16) Male infertility factor non-PCOS (n=19) Unexplained infertility non-PCOS (n=19)	29.4 ± 4.8 28.8 ± 3.3 29.1 ± 6.9	24.5 ± 6.4 25.4 ± 4.9 23.5 ± 3.5	IVF GnRH agonist protocol, rFSH	**FF:** AMH **IVF outcomes:** total gonadotropin dose, stimulation duration, retrieved oocytes (n), MII oocytes (n), 2PN (n), embryo (n)	FF AMH levels were not significantly different between the three groups as well as between pregnant and non-pregnant groups. FF AMH was negatively correlated with FSH (day 3) and gonadotropin dose and positively correlated* with oocyte, 2PN and embryo counts, in PCOS group.
Garruti, G. 2014 [49]	Prospective cohort	PCOS (n=16) Ovulatory non-PCOS (n=10)	32.1 ± 3.6 34.4 ± 3.1	22.6 ± 2.6 22.6 ± 2.6	ICSI GnRH agonist protocol, rFSH	**FF:** leptin and visfatin **Serum:** free TT, Δ4-androstenedione, SHBG, DHT, DHEA, DHEAS, HDL-C, LDL-C, triglycerides, 17β-E_2_, insulin levels, leptin and visfatin. **ICSI outcomes**: Follicles (size), MII oocytes (n), fertilization rate (%), embryos (I°) pregnancy rate (%)	**PCOS:** ↓leptin Positive correlation between BMI and FF-leptin in women with PCOS. FF-visfatin levels were not significantly different between groups.
Inal, H. A. 2016 [63]	Prospective cross-sectional	PCOS (n=60) Ovulatory non-PCOS (n=60)	29.4 ± 3.7 30.0 ± 3.7 30.3 ± 4.0 31.6 ± 4.0	BMI<25 BMI≥25 BMI<25 BMI≥25	IVF GnRH agonist protocol, rFSH	**FF:** adiponectin and ghrelin. **IVF outcomes:** clinical pregnancy	PCOS: ↓adiponectin FF adiponectin levels were lower* in the lean and overweight PCOS than in lean non-PCOS. There was no significant difference in the FF ghrelin levels between the groups.
Chen, 2017 [58]	Prospective cohort	PCOS (n=59) Ovulatory non-PCOS (n=120)	29.1 ± 3.5 30.3 ± 3.9	22.2 ± 3.2 21.4 ± 2.9	IVF/ICSI PCOS group: GnRH agonist (n=30) or GnRH antagonist (n=29), rFSH Control group: GnRH agonist protocol, rFSH	**FF:** AMH **Serum:** AMH **IVF/ICSI outcomes:** AFC, peak E_2_, oocytes retrieved (n), MII oocytes (n), oocyte maturity rate (%), FR (%), good-quality embryos (n), 2PN (n), good-quality embryo rate (%), pregnancy rate (%), abortion rate (%), implantation rate (%)	**PCOS:** ↑AMH FF AMH positively correlated* with AFC in PCOS group. FF AMH levels were independent predictor of oocyte number.
Jaschke, N. 2018 [67]	Prospective cohort	PCOS (n=16) Ovulatory non-PCOS (n=43)	34.7 ± 5.0 34.7 ± 5.0	29.3 ± 4.0 23.7 ± 5.1	No information	**FF:** β-endorphin **Serum:** β-endorphin **IVF outcomes:** oocytes (n), MII oocytes retrieved (n)	There was no difference in β-endorphin levels between PCOS and non-PCOS women. FF β-endorphin was positively correlated with MII oocytes retrieved. FF β-endorphin levels positively correlated with TT levels.
Liu, X. H. 2019 [57]	Case-control	PCOS (n=52) Ovulatory non-PCOS (n=61)	29.7 ± 0.5 30.6 ± 0.4	27.1 ± 0.6 22.6 ± 0.4	IVF GnRH antagonist protocol, rFSH	**FF:** AMH and SCF **Serum:** AMH and SCF **IVF outcomes:** retrieved oocytes (n), MII oocytes (n), 2PN fertilization (n), high-quality embryos (n)	**PCOS:** ↑AMH ↓SCF In the control group, the basal AMH was negatively correlated with Gn dosage and positive correlated* with controlled ovarian hyperstimulation outcomes (number of retrieved oocytes, MII oocytes, and 2PN fertilization).
Bousmpoula, A. 2019 [71]	Cohort	PCOS (n=70) BMI<25 =35 BMI>25=35 Ovulatory non-PCOS (n=70) BMI<25 =35 BMI>25=35	31.9 ± 4.3 32.2 ± 3.9 35.1 ± 4.5 35.3 ± 4.5	22.2 ± 1.1 26.8 ± 1.4 22.3 ± 1.2 26.6 ± 1.3	No information	**FF:** irisin **Serum:** irisin, insulin, glucose, E_2_, LH, FSH, TT, SHBG, cholesterol, HDL-C, LDL-C, triglycerides, apolipoprotein A1, apolipoprotein B, lipoprotein(a) and homocysteine **IVF outcomes:** oocytes (n), Fertilized oocytes (n), Transferred embryos (n), Pregnancy [n (%)]	In overweight PCOS women, both serum and FF irisin levels were positively correlated to FSH and LH. In non-overweight controls, FF irisin levels were negatively correlated with FAI and testosterone levels. In non-overweight PCOS patients, FF irisin levels were positively correlated with homocysteine. Irisin correlated positively with BMI, dyslipidemia and the number of oocytes retrieved and fertilized.
Hu, K. L. 2019 [50]	cohort	PCOS (n=49) Ovulatory non-PCOS (n=52)	29.6 ± 3.6 30.6 ± 4.1	24.5 ± 3.8 24.3 ± 3.5	IVF/ICSI luteal-phase GnRH agonist or follicular-phase GnRH antagonist	**FF:** KISS1, KISS1R **Serum:** AMH, FSH, TT, LH, E_2_, PRL, P_4_, and A **IVF/ICSI outcomes:** oocyte retrieved (n), fertilized egg (n), 2PN (n), day 3 viable embryo (n), transferred embryo (n)	**PCOS:** ↑KISS1 and KISS1R KISS1 expression in human granulosa lutein cells was positively correlated with KISS1R expression in women without PCOS, but not in women with PCOS. KISS1 expression in GCs was positively correlated with the serum levels of AMH.
Liu, Y. 2019 [53]	Case-control	PCOS (n=261) Ovulatory non-PCOS (n=217)	29.8 ± 3.5 29.6 ± 3.2	< 25=106, 25-30=98, > 30=57 < 25=147, 25-30=60, > 30=10	ICSI PCOS group: GnRH agonist long protocol, FSH and rFSH	**FF:** E_2,_ TT **Serum:** E_2,_ TT, SHBG, P_4_, PRL **IVF/ ICSI outcomes:** Endometrial thickness, retrieved oocytes and embryos (n), FR (%), Pregnancy rate (%), Abortion rate (%), Transplant rejection rate (%)	**PCOS:** ↑E_2_, TT In comparison with the control group, the levels of E_2_ and TT in serum as well as testosterone level in FF were found to be significantly increased in patients of the PCOS group on the day of HCG injection, while the serum level of SHBG was found to be markedly downregulated.
Gao, H. 2021 [72]	Prospective cohort	PCOS (n=32) Ovulatory non-PCOS (n=28)	28.9 ± 3.4 29.5 ± 3.5	22.2 ± 3.0 20.2 ± 2.5	IVF GnRH agonist protocol, rFSH	**FF:** TSH **Serum:** TSH **IVF outcomes:** oocyte maturation rate (%), FR (%), cleavage rate (%), high quality embryo rate (%), pregnancy rate (%)	**PCOS:** ↑TSH TSHR was upregulated in PCOS. Serum and FF TSH levels were negatively correlated with oocyte maturation and fertilization rates in PCOS.
Zhang, C. 2021 [52]	Cohort	PCOS (n=90) Ovulatory non-PCOS (n=100)	29.4 ± 3.8 30.2 ± 5.1	22.8 ± 1.6 21.7 ± 1.9	IVF GnRH agonist protocol	**FF:** β-endorphin levels **Serum:** β-endorphin levels, E_2_, FSH, LH, TT, prolactin, AMH, fasting insulin, and fasting blood glucose **IVF outcomes:** MII oocytes (n), high-quality embryos (n), fertilized oocytes (n), rate of normal fertilization (%), rate of oocyte differentiation, rate of D3 high-quality embryo (%), pregnancy (%) live birth (%), abortion (%)	**PCOS:** ↑β-endorphin AMH, serum and FF β-endorphin were positively correlated with oocyte quality. Serum and FF β-endorphin had high sensitivity and specificity to predict pregnancy and live birth.

*A* androstenedione, *AFC* antral follicle count, *AMH* antimüllerian hormone, *BMI* body mass index, *DHEA* dehydroepiandrosterone, *DHEAS* dehydroepiandrosterone sulfate, *DHT* dihydrotestosterone, *E*_*2*_ oestradiol, *FAI* free androgen index, *FF* follicular fluid, *FR* fertilization rate, *GnRH* gonadotropin-releasing hormone, *GCs* granulosa cells, *hMG* human menopausal gonadotrophins, *ICS*I intracytoplasmic sperm injection, *IVF* *in vitro* fertilization, *KISS1* kisspeptin 1, *LH* luteinizing hormone, *MI* metaphase I, *MII* metaphase II, *mRNA* messenger ribonucleic acid, *n* number, *P*_*4*_ progesterone, *PCOS* polycystic ovarian syndrome, *2PN* two pronuclei (fertilized oocytes), *p-STAT3* signal transducer and activator of transcription 3, *PRL* prolactin, *rFSH* recombinant follicle stimulating hormone, *SCF* stem cell factor, *SHBG* sex hormone-binding globulin, *TT* testosterone, *TSH* thyroid stimulating hormone

**Table 5:** Summary of studies comparing other molecules in follicular fluid of PCOS and non-PCOS women

**First Author** **Publication Year**	**Experimental Design**	**Population**			**IVF/ICSI Treatment** **Ovarian Induction**	**Outcome Measures**	**Results**
		**Groups**	**Age (years)**	**BMI (kg/m**^**2**^**)**			
**Other molecules**							
Niu, Z. 2014 [85]		PCOS (n=55) BMI < 25 (n=30) BMI > 30 (n=25) Ovulatory non-PCOS (n=63) BMI < 25 (n=38) BMI > 30 (n=25)	31.2 ± 3.7 30.9 ± 3.8 30.6 ± 3.5 30.9 ± 3.6	22.9 ± 3.1 32.4 ± 2.4 22.1 ± 2.8 32.9 ± 2.2	IVF GnRH antagonist protocol, rFSH, and/or HMG	**FF:** free fatty acids. **Serum:** FSH, LH, E_2_, TT, insulin, TC, TG, LDL, HDL, free fatty acids **IVF outcomes:** Embryo fragmentation, Blastomere score	**PCOS: ↑**palmitic acid, oleic acid The embryo fragmentation score was positively correlated* with the oleic acid concentration in women with PCOS both with and without obesity.
Timur, H. 2015 [86]	Cohort	PCOS (n=41) Ovulatory non-PCOS (n=40)	27.5 ± 3.4 28.1 ± 2.4	24.9 ± 1.7 24.6 ± 1.3	ICSI GnRH agonist protocol, rFSH	**FF:** amyloid A levels **Serum:** amyloid A levels **ICSI outcomes:** Endometrial thickness (mm), retrieved oocytes (n), mature oocytes (n), FR (%), Patients with grade 1 and 2 embryos (%), Live birth rate (%), Clinical pregnancy rate (%), Abortion rate (%)	No significant difference was found between the two groups regarding the serum and FF amyloid A protein levels on the day of oocyte retrieval.
Zhang, Y. 2017 [77]	Cohort	PCOS (n=15) Ovulatory non-PCOS (n=36)	27.6 ± 3.0 31.9 ± 3.8	No information	No information	**FF:** Metabolic profile **Outcomes:** 2PN FR (%), Cleavage rate (%), Top-quality embryos rate (%)	**PCOS:** ↑glycoprotein, acetate and cholesterol ↓lactic acid, glutamine, pyruvate and alanine. A positive correlation* was observed between 2PN fertilization rate and acetoacetate, acetate, 3-hydroxuybutyrate, glycoprotein and formic acid and a negative correlation was observed between glucose and fertilization results, in women with PCOS. Acetic acid level was negatively correlated with the cleavage results in controls; however, it was positively correlated with 2PN fertilization rate in PCOS. Creatine, leucine, isoleucine, and tyrosine were negatively correlated* with embryos quality in all samples. Histidine exhibited highly negative correlation and glutamine presented highly positive correlation with embryos top-quality rate respectively, in PCOS.
Tola, E. N. 2017 [83]	Prospective cohort	PCOS (n=21) Ovulatory non-PCOS (n=22)	29.2 ± 3.7 29.1 ± 3.6	27.9 ± 5.9 25.2 ± 5.4	ICSI GnRH antagonist protocol, rFSH	**FF:** ADAMTS-1 and aggrecan **ICSI outcomes:** oocytes retrieved (n), MI and MII oocytes (n and %), fertilization rate (%), implantation rate (%)	**PCOS:** ↑ADAMTS-1 and aggrecan Higher ADAMTS-1 FF levels were related to increased implantation in PCOS.
Inal, Z. O. 2018 [87]	Prospective cross-sectional	PCOS (n=80) Ovulatory non-PCOS women (n=80)	27.6 ± 4.1 28.9 ± 4.7	26.3 ± 4.1 25.0 ± 3.4	ICSI GnRH agonist protocol, rFSH	**FF:** Sfrp-5 **Serum:** Sfrp-5 **ICSI outcomes:** retrieved and MII oocytes (n), FR (%), embryo quality (%), clinical pregnancy rate (%), live birth rate (%)	**PCOS: ↑**Sfrp-5 Serum and FF Sfrp-5 levels were positively correlated* with fasting insulin and negatively correlated* with inflammatory markers (CRP and neutrophil count). Serum and FF Sfrp-5 levels were not associated with the clinical pregnancy rate.
Butler, A. E. 2019 [73]	Prospective cohort	PCOS (n=29) Ovulatory non-PCOS (n=30)	30.9 ± 4.8 32.6 ± 4.7	26.0 ± 3.8 25.5 ± 3.6	IVF GnRH agonist protocol, rFSH	**FF:** 176 miRNAs **Serum:** TT, SHBG, AMH, FAI, HOMA-IR **IVF outcomes:** endometrium thickness oocytes retrieved (n), fertilization rate (%), cleavage and blastocyst stages embryos (n), G3D3 embryos (n), clinical pregnancy (n)	Of 176 miRNAs detected in FF, 29 differed* between normal women and PCOS. The top 7 of these were miR-381-3p, miR-199b-5p, miR-93-3p, miR-361-3p, miR-127-3p, miR-382-5p, miR-425-3p. In the PCOS group, miR-382-5p positively correlated* with age and negatively* with FAI, miR-199b-5p negatively correlated* with AMH and miR-93-3p positively correlated* with CRP. In controls, miR-127-3p, miR382-5p and miR425-3p positively correlated* with the fertilisation rate; miR-127-3p negatively correlated* with insulin resistance, and miR-381-3p negatively correlated* with FAI.
Sun, Y. 2019 [88]	Cohort study	PCOS (n=89) Ovulatory non-PCOS (n=114) Fallopian tube obstruction (FTO) non-PCOS (n=145)	29.8 ± 4.1 32.4 ± 4.0 32.2 ± 4.8	21.8 ± 2.8 21.0 ± 3.0 21.6 ± 2.5	ICSI GnRH agonist long protocol, rFSH and hMG	**FF:** Levels of 22 trace elements **Serum:** FSH, LH, TT, E_2_, PRL and AMH **ICSI outcomes:** Endometrial thickness (mm), retrieved oocytes (n), FR (%), cleavage rate (%), High-quality embryo rate (%)	**PCOS:** ↑copper Higher FF copper levels were associated with a higher* number of retrievable oocytes in the PCOS group but a lower rate of high-quality embryos. FF copper levels were positively correlated with TT and P_4_ levels. Cultured human GCs overexposed to copper showed increased E_2_ secretion and decreased TT levels. It was also found an increase in CYP19A1 and HSD3b mRNA expression.

*ADAMTS-1* a disintegrin-like and metalloproteinase with thrombospondin type motifs 1, *AMH* antimüllerian hormone, *BMI* body mass index, *CRP* C-reactive protein, *CYP19A1* cytochrome P450 family 19 subfamily A member 1, *E*_*2*_ oestradiol, *FAI* free androgen index, *FF* follicular fluid, 21, *FR* fertilization rate, *GnRH* gonadotropin-releasing hormone, *GCs* granulosa cells, *G3D3* top quality embryos on day 3, *HDL* high-density lipoprotein, *hMG* human menopausal gonadotrophins, *HOMA-IR* homeostasis model assessment estimate of insulin resistance, *ICSI* intracytoplasmic sperm injection, *IVF* *in vitro* fertilization, *LDL* low-density lipoprotein, LH luteinizing hormone, *MI* metaphase I, *MII* metaphase II, *miRNA* micro ribonucleic acid, *mRNA* messenger ribonucleic acid, *n* number, *P*_*4*_ progesterone, *PCOS* polycystic ovarian syndrome, *2PN* two pronuclei (fertilized oocytes), *rFSH* recombinant follicle stimulating hormone, *Sfrp-5* secreted frizzle-related protein-5, *SHBG* sex hormone-binding globulin, *TC* total cholesterol, *TG* triglycerides, *TT* total testosterone

## Abbreviations

ADAMTS-1: A disintegrin-like and metalloproteinase with thrombospondin type motifs-1
AMH: Anti-Müllerian hormone
ART: Assisted reproductive technology
CAT: Catalase
CRP: C-reactive protein
FF: Follicular fluid
FGF: Fibroblast growth factor
GCs: Granulosa cells
G-CSF: Granulocyte colony-stimulating factor
GDF-8: Growth differentiation factor-8
GPx: Glutathione peroxidase
HGF: Hepatocyte growth factor
IVF: *in vitro* fertilization
LIF: Leukaemia inhibitory factor
LPO: Lipid peroxidation
MDA: Malondialdehyde
PCOS: Polycystic ovary syndrome
PGRN: Progranulin
PlGF: Placental growth factor
PRISMA: Preferred Reporting Items for Systematic Reviews and Meta-Analyses
ROS: Reactive oxygen species
Sfrp-5: Secreted frizzle-related protein-5
SOD: Superoxide dismutase
sRAGE: Soluble receptor for advanced glycation endproducts
TAC: Total antioxidant capacity
TNF-α: Tumour necrosis factor
TSH: Thyroid-stimulating hormone
VEGF: Vascular endothelial growth factor

## References

[1] Yildiz BO (2012). Prevalence, phenotype and cardiometabolic risk of polycystic ovary syndrome under different diagnostic criteria. Hum Reprod.

[2] Balen AH (2016). The management of anovulatory infertility in women with polycystic ovary syndrome: an analysis of the evidence to support the development of global WHO guidance. Hum Reprod Update.

[3] Escobar-Morreale HF (2018). Polycystic ovary syndrome: definition, aetiology, diagnosis and treatment. Nat Rev Endocrinol.

[4] Franks S (1995). Polycystic Ovary Syndrome..

[5] Teede HJ (2018). Recommendations from the international evidence-based guideline for the assessment and management of polycystic ovary syndrome. Fertility and Sterility.

[6] Artimani T (2018). Evaluation of pro-oxidant-antioxidant balance (PAB) and its association with inflammatory cytokines in polycystic ovary syndrome (PCOS). Gynecological Endocrinology.

[7] Piomboni P (2014). Protein modification as oxidative stress marker in follicular fluid from women with polycystic ovary syndrome: the effect of inositol and metformin. J Assist Reprod Genet.

[8] Rajani S (2012). Assessment of oocyte quality in polycystic ovarian syndrome and endometriosis by spindle imaging and reactive oxygen species levels in follicular fluid and its relationship with IVF-ET outcome. J Hum Reprod Sci.

[9] Yilmaz N (2016). Follicular fluid total antioxidant capacity levels in PCOS. J Obstet Gynaecol.

[10] Qiao J, Feng HL (2011). Extra- and intra-ovarian factors in polycystic ovary syndrome: impact on oocyte maturation and embryo developmental competence. Hum Reprod Update.

[11] Piltonen TT (2016). Polycystic ovary syndrome: Endometrial markers. Best Pract Res Clin Obstet Gynaecol.

[12] Da Broi MG (2018). Influence of follicular fluid and cumulus cells on oocyte quality: clinical implications. Journal of Assisted Reproduction and Genetics.

[13] Palomba S, Daolio J, La Sala GB (2017). Oocyte Competence in Women with Polycystic Ovary Syndrome. Trends in Endocrinology and Metabolism.

[14] Edwards RG (1974). Follicular fluid. J Reprod Fertil.

[15] Jančar N (2007). Effect of apoptosis and reactive oxygen species productionin human granulosa cells on oocyte fertilizationand blastocyst development. Journal of Assisted Reproduction and Genetics.

[16] Zhao Y (2022). Exosomal miR-143-3p derived from follicular fluid promotes granulosa cell apoptosis by targeting BMPR1A in polycystic ovary syndrome. Scientific reports.

[17] Ambekar AS (2015). Proteomics of follicular fluid from women with polycystic ovary syndrome suggests molecular defects in follicular development. J Clin Endocrinol Metab.

[18] Sun Z (2019). Identification of potential metabolic biomarkers of polycystic ovary syndrome in follicular fluid by SWATH mass spectrometry. Reproductive Biology and Endocrinology.

[19] Page MJ (2021). The PRISMA 2020 statement: An updated guideline for reporting systematic reviews. PLOS Medicine.

[20] Shamseer L (2015). Preferred reporting items for systematic review and meta-analysis protocols (PRISMA-P) 2015: elaboration and explanation. BMJ : British Medical Journal.

[21] Artimani T (2018). Evaluation of pro-oxidant-antioxidant balance (PAB) and its association with inflammatory cytokines in polycystic ovary syndrome (PCOS). Gynecol Endocrinol.

[22] Chattopadhayay R (2010). Effect of follicular fluid oxidative stress on meiotic spindle formation in infertile women with polycystic ovarian syndrome. Gynecol Obstet Invest.

[23] Naigaonkar A (2021). Altered redox status may contribute to aberrant folliculogenesis and poor reproductive outcomes in women with polycystic ovary syndrome. J Assist Reprod Genet.

[24] Gongadashetti K (2021). Follicular fluid oxidative stress biomarkers and ART outcomes in PCOS women undergoing in vitro fertilization: A cross-sectional study. Int J Reprod Biomed.

[25] Seleem AK (2014). Superoxide dismutase in polycystic ovary syndrome patients undergoing intracytoplasmic sperm injection. J Assist Reprod Genet.

[26] Tola EN (2018). The Role of Follicular Fluid Thiol/Disulphide Homeostasis in Polycystic Ovary Syndrome. Balkan Med J.

[27] Wang B (2016). Follicular fluid soluble receptor for advanced glycation endproducts (sRAGE): a potential protective role in polycystic ovary syndrome. J Assist Reprod Genet.

[28] Wang Y (2019). High concentration of chemerin caused by ovarian hyperandrogenism may lead to poor IVF outcome in polycystic ovary syndrome: a pilot study. Gynecol Endocrinol.

[29] Zhang H (2020). IL-18 and IL-18 binding protein concentration in ovarian follicular fluid of women with unexplained infertility to PCOS during in vitro fertilization. J Reprod Immunol.

[30] Niu Z (2017). Follicular fluid cytokine composition and oocyte quality of polycystic ovary syndrome patients with metabolic syndrome undergoing in vitro fertilization. Cytokine.

[31] Zhou D (2017). Increased expression of PGRN protein in follicular fluid and mRNA in granulosa cells in overweight patients with polycystic ovary syndrome. Eur J Obstet Gynecol Reprod Biol.

[32] Ozörnek MH, et al. Epidermal growth factor and leukemia inhibitory factor levels in follicular fluid. Association with *in vitro* fertilization outcome*.* J Reprod Med. 1999;44(4):367-369. 10319308

[33] Li Z (2018). Leukaemia inhibitory factor in serum and follicular fluid of women with polycystic ovary syndrome and its correlation with IVF outcome. Reprod Biomed Online.

[34] Maillard-Lefebvre H (2009). Soluble receptor for advanced glycation end products: a new biomarker in diagnosis and prognosis of chronic inflammatory diseases. Rheumatology.

[35] Garg D (2017). Correlation between follicular fluid levels of sRAGE and vitamin D in women with PCOS. J Assist Reprod Genet.

[36] Wang B (2017). Decreased levels of sRAGE in follicular fluid from patients with PCOS. Reproduction.

[37] Kahyaoglu I (2015). Granulocyte colony-stimulating factor: A relation between serum and follicular fluid levels and in-vitro fertilization outcome in patients with polycystic ovary syndrome. Cytokine.

[38] Kudsy M, Alhalabi M, Al-Quobaili F (2016). Follicular fluid Vascular Endothelial Growth Factor (VEGF) could be a predictor for pregnancy outcome in normo-responders and polycystic ovary syndrome women undergoing IVF/ICSI treatment cycles. Middle East Fertility Society Journal.

[39] Savchev SI (2010). Follicular fluid-specific distribution of vascular endothelial growth factor isoforms and sFlt-1 in patients undergoing IVF and their correlation with treatment outcomes. Reprod Sci.

[40] Tal R (2014). Follicular fluid placental growth factor is increased in polycystic ovarian syndrome: correlation with ovarian stimulation. Reprod Biol Endocrinol.

[41] Fang L (2020). High GDF-8 in follicular fluid is associated with a low pregnancy rate in IVF patients with PCOS. Reproduction.

[42] Uzumcu M (2006). Immunolocalization of the hepatocyte growth factor (HGF) system in the rat ovary and the anti-apoptotic effect of HGF in rat ovarian granulosa cells in vitro. Reproduction.

[43] Guglielmo MC (2011). The effect of hepatocyte growth factor on the initial stages of mouse follicle development. Journal of Cellular Physiology.

[44] Şahin N (2013). The levels of hepatocyte growth factor in serum and follicular fluid and the expression of c-Met in granulosa cells in patients with polycystic ovary syndrome. Fertility and Sterility.

[45] Buratini J (2007). Expression and Function of Fibroblast Growth Factor 10 and Its Receptor, Fibroblast Growth Factor Receptor 2B, in Bovine Follicles1. Biology of Reproduction.

[46] Liu Y (2018). Intrafollicular fibroblast growth factor 13 in polycystic ovary syndrome: relationship with androgen levels and oocyte developmental competence. Journal of Ovarian Research.

[47] Şahin N (2013). The levels of hepatocyte growth factor in serum and follicular fluid and the expression of c-Met in granulosa cells in patients with polycystic ovary syndrome. Fertil Steril.

[48] Liu Y (2018). Intrafollicular fibroblast growth factor 13 in polycystic ovary syndrome: relationship with androgen levels and oocyte developmental competence. J Ovarian Res.

[49] Garruti G (2014). Association between follicular fluid leptin and serum insulin levels in nonoverweight women with polycystic ovary syndrome. Biomed Res Int.

[50] Hu KL (2019). Increased Expression of KISS1 and KISS1 Receptor in Human Granulosa Lutein Cells-Potential Pathogenesis of Polycystic Ovary Syndrome. Reprod Sci.

[51] Li MG (2007). Association of serum and follicular fluid leptin concentrations with granulosa cell phosphorylated signal transducer and activator of transcription 3 expression in fertile patients with polycystic ovarian syndrome. J Clin Endocrinol Metab.

[52] Zhang C (2021). β-Edorphin predict pregnancy outcome of PCOS and DOR women after IVF-ET. Arch Gynecol Obstet.

[53] Liu Y (2019). Effect of sex hormone-binding globulin polymorphisms on the outcome of in vitro fertilization-embryo transfer for polycystic ovary syndrome patients: A case-control study. J Cell Biochem.

[54] Dewailly D (2020). Role of Anti-Müllerian Hormone in the Pathogenesis of Polycystic Ovary Syndrome. Front Endocrinol (Lausanne).

[55] Shaaban Z (2019). Pathophysiological mechanisms of gonadotropins- and steroid hormones-related genes in etiology of polycystic ovary syndrome. Iran J Basic Med Sci.

[56] Parco S (2011). Serum anti-Müllerian hormone as a predictive marker of polycystic ovarian syndrome. Int J Gen Med.

[57] Liu XH, Wu XH, Yang S (2019). Changes and correlations of anti-Müllerian hormone and stem-cell factors in different ovarian reserve patients during GnRH-antagonist protocol and the effects on controlled ovarian hyperstimulation outcomes. Arch Gynecol Obstet.

[58] Chen Y (2017). Predicting the outcome of different protocols of in vitro fertilization with anti-Muüllerian hormone levels in patients with polycystic ovary syndrome. J Int Med Res.

[59] Yilmaz N (2012). The effect of follicular antimullerian hormone levels of non-obese, non-hyperandrogenemic polycystic ovary syndrome patients on assisted reproduction outcome. Gynecol Endocrinol.

[60] Zeydabadi Nejad S, Ramezani Tehrani F,Zadeh-Vakili A. The Role of Kisspeptin in Female Reproduction*.* Int J Endocrinol Metab. 2017;15(3):e44337. 10.5812/ijem.44337 PMC5702467 29201072

[61] Polak AM (2020). The Association of Serum Levels of Leptin and Ghrelin with the Dietary Fat Content in Non-Obese Women with Polycystic Ovary Syndrome..

[62] Maffei M (1995). Leptin levels in human and rodent: Measurement of plasma leptin and ob RNA in obese and weight-reduced subjects. Nature Medicine.

[63] Inal HA (2016). The impact of follicular fluid adiponectin and ghrelin levels based on BMI on IVF outcomes in PCOS. J Endocrinol Invest.

[64] Bousmpoula A (2019). Serum and follicular fluid irisin levels in women with polycystic ovaries undergoing ovarian stimulation: correlation with insulin resistance and lipoprotein lipid profiles. Gynecological Endocrinology.

[65] Cumming DC (1984). EVIDENCE FOR DECREASED ENDOGENOUS DOPAMINE AND OPIOID INHIBITORY INFLUENCES ON LH SECRETION IN POLYCYSTIC OVARY SYNDROME. Clinical Endocrinology.

[66] Guido M (1998). Involvement of Ovarian Steroids in the Opioid-Mediated Reduction of Insulin Secretion in Hyperinsulinemic Patients with Polycystic Ovary Syndrome. The Journal of Clinical Endocrinology & Metabolism.

[67] Jaschke N (2018). Beta endorphin in serum and follicular fluid of PCOS- and non-PCOS women. Arch Gynecol Obstet.

[68] Duran C (2015). Frequency of nodular goiter and autoimmune thyroid disease in patients with polycystic ovary syndrome. Endocrine.

[69] Gao H (2021). Thyroid-stimulating hormone level is negatively associated with fertilization rate in patients with polycystic ovary syndrome undergoing in vitro fertilization. International Journal of Gynecology & Obstetrics.

[70] Nafiye Y (2010). The effect of serum and intrafollicular insulin resistance parameters and homocysteine levels of nonobese, nonhyperandrogenemic polycystic ovary syndrome patients on in vitro fertilization outcome. Fertil Steril.

[71] Bousmpoula A (2019). Serum and follicular fluid irisin levels in women with polycystic ovaries undergoing ovarian stimulation: correlation with insulin resistance and lipoprotein lipid profiles. Gynecol Endocrinol.

[72] Gao H (2021). Thyroid-stimulating hormone level is negatively associated with fertilization rate in patients with polycystic ovary syndrome undergoing in vitro fertilization. Int J Gynaecol Obstet.

[73] Butler AE (2019). Expression of microRNA in follicular fluid in women with and without PCOS. Sci Rep.

[74] Wang Y (2019). High concentration of chemerin caused by ovarian hyperandrogenism may lead to poor IVF outcome in polycystic ovary syndrome: a pilot study. Gynecological Endocrinology.

[75] Duplus E, Forest C (2002). Is there a single mechanism for fatty acid regulation of gene transcription?. Biochemical Pharmacology.

[76] Niu Z (2014). Associations Between Insulin Resistance, Free Fatty Acids, and Oocyte Quality in Polycystic Ovary Syndrome During In Vitro Fertilization. The Journal of Clinical Endocrinology & Metabolism.

[77] Zhang Y (2017). Follicular metabolic changes and effects on oocyte quality in polycystic ovary syndrome patients. Oncotarget.

[78] Ouchi N (2010). Sfrp5 Is an Anti-Inflammatory Adipokine That Modulates Metabolic Dysfunction in Obesity. Science.

[79] Carstensen M (2013). Sfrp5 correlates with insulin resistance and oxidative stress. European Journal of Clinical Investigation.

[80] Hu Z, Deng H, Qu H (2013). Plasma SFRP5 levels are decreased in Chinese subjects with obesity and type 2 diabetes and negatively correlated with parameters of insulin resistance. Diabetes Research and Clinical Practice.

[81] Inal ZO, Inal HA, Erdem S (2018). The effect of serum and follicular fluid secreted frizzle-related protein-5 on in vitro fertilization outcomes in patients with polycystic ovary syndrome. Molecular Biology Reports.

[82] Brown HM (2010). ADAMTS1 Cleavage of Versican Mediates Essential Structural Remodeling of the Ovarian Follicle and Cumulus-Oocyte Matrix During Ovulation in Mice1. Biology of Reproduction.

[83] Tola EN (2017). Follicular ADAMTS-1 and aggrecan levels in polycystic ovary syndrome. J Assist Reprod Genet.

[84] Timur H (2015). The effect of serum and follicular fluid amyloid-associated protein levels on in vitro fertilization outcome in patients with polycystic ovary syndrome. Journal of Assisted Reproduction and Genetics.

[85] Niu Z (2014). Associations between insulin resistance, free fatty acids, and oocyte quality in polycystic ovary syndrome during in vitro fertilization. J Clin Endocrinol Metab.

[86] Timur H (2015). The effect of serum and follicular fluid amyloid-associated protein levels on in vitro fertilization outcome in patients with polycystic ovary syndrome. J Assist Reprod Genet.

[87] Inal ZO, Inal HA, Erdem S (2018). The effect of serum and follicular fluid secreted frizzle-related protein-5 on in vitro fertilization outcomes in patients with polycystic ovary syndrome. Mol Biol Rep.

[88] Sun Y (2019). High copper levels in follicular fluid affect follicle development in polycystic ovary syndrome patients: Population-based and in vitro studies. Toxicol Appl Pharmacol.

[89] McCartney CR, Campbell RE (2020). Abnormal GnRH Pulsatility in Polycystic Ovary Syndrome: Recent Insights. Curr Opin Endocr Metab Res.

[90] Cate RL (1986). Isolation of the bovine and human genes for Müllerian inhibiting substance and expression of the human gene in animal cells. Cell.

[91] Moolhuijsen LME, Visser JA (2020). Anti-Müllerian Hormone and Ovarian Reserve: Update on Assessing Ovarian Function. The Journal of Clinical Endocrinology & Metabolism.

[92] Patel S (2018). Polycystic ovary syndrome (PCOS), an inflammatory, systemic, lifestyle endocrinopathy. The Journal of Steroid Biochemistry and Molecular Biology.

[93] Lu J (2018). A novel and compact review on the role of oxidative stress in female reproduction. Reproductive Biology and Endocrinology.

[94] Ayala A, Muñoz MF, Argüelles S (2014). Lipid peroxidation: production, metabolism, and signaling mechanisms of malondialdehyde and 4-hydroxy-2-nonenal. Oxid Med Cell Longev.

[95] Freitas C (2017). Follicular Fluid redox involvement for ovarian follicle growth. Journal of Ovarian Research.

[96] Popko K, et al. Proinflammatory cytokines Il-6 and TNF-α and the development of inflammation in obese subjects*.* Eur J Med Res. 2010;15 Suppl 2(Suppl 2):120-2. 10.1186/2047-783X-15-S2-120 PMC4360270 21147638

[97] Vural P (2010). Tumor necrosis factor α (−308), interleukin-6 (−174) and interleukin-10 (−1082) gene polymorphisms in polycystic ovary syndrome. European Journal of Obstetrics & Gynecology and Reproductive Biology.

[98] Xie J (2013). Cellular signalling of the receptor for advanced glycation end products (RAGE). Cellular Signalling.

[99] Merhi Z (2014). Advanced glycation end products and their relevance in female reproduction. Human Reproduction.

[100] Uribarri J (2015). Dietary Advanced Glycation End Products and Their Role in Health and Disease. Advances in Nutrition.

[101] Lam PM, Haines C (2005). Vascular endothelial growth factor plays more than an angiogenic role in the female reproductive system. Fertility and Sterility.

[102] Pepper MS (1991). Vascular endothelial growth factor (VEGF) induces plasminogen activators and plasminogen activator inhibitor-1 in microvascular endothelial cells. Biochemical and Biophysical Research Communications.

[103] Peitsidis P, Agrawal R (2010). Role of vascular endothelial growth factor in women with PCO and PCOS: a systematic review. Reproductive BioMedicine Online.

[104] Stanek MB (2007). Insulin and Insulin-Like Growth Factor Stimulation of Vascular Endothelial Growth Factor Production by Luteinized Granulosa Cells: Comparison between Polycystic Ovarian Syndrome (PCOS) and Non-PCOS Women. The Journal of Clinical Endocrinology & Metabolism.

[105] Teede H, Deeks A, Moran L (2010). Polycystic ovary syndrome: a complex condition with psychological, reproductive and metabolic manifestations that impacts on health across the lifespan. BMC Med.

[106] Schultz DR, Arnold PI (1990). Properties of four acute phase proteins: C-reactive protein, serum amyloid A protein, alpha 1-acid glycoprotein, and fibrinogen. Semin Arthritis Rheum.

